# The cholesterol 24-hydroxylase CYP46A1 promotes α-synuclein pathology in Parkinson’s disease

**DOI:** 10.1371/journal.pbio.3002974

**Published:** 2025-02-18

**Authors:** Lijun Dai, Jiannan Wang, Lanxia Meng, Xingyu Zhang, Tingting Xiao, Min Deng, Guiqin Chen, Jing Xiong, Wei Ke, Zhengyuan Hong, Lihong Bu, Zhentao Zhang

**Affiliations:** 1 Department of Neurology, Renmin Hospital of Wuhan University, Wuhan, China; 2 PET-CT/MRI Center, Molecular Imaging Center, Renmin Hospital of Wuhan University, Wuhan, China; 3 TaiKang Center for Life and Medical Sciences, Wuhan University, Wuhan, China; Stony Brook University Medical Center: Stony Brook University Hospital, UNITED STATES OF AMERICA

## Abstract

Parkinson’s disease (PD) is a neurodegenerative disease characterized by the death of dopaminergic neurons in the substantia nigra and the formation of Lewy bodies that are composed of aggregated α-synuclein (α-Syn). However, the factors that regulate α-Syn pathology and nigrostriatal dopaminergic degeneration remain poorly understood. Previous studies demonstrate cholesterol 24-hydroxylase (CYP46A1) increases the risk for PD. Moreover, 24-hydroxycholesterol (24-OHC), a brain-specific oxysterol that is catalyzed by CYP46A1, is elevated in the cerebrospinal fluid of PD patients. Herein, we show that the levels of CYP46A1 and 24-OHC are elevated in PD patients and increase with age in a mouse model. Overexpression of CYP46A1 intensifies α-Syn pathology, whereas genetic removal of CYP46A1 attenuates α-Syn neurotoxicity and nigrostriatal dopaminergic degeneration in the brain. Moreover, supplementation with exogenous 24-OHC exacerbates the mitochondrial dysfunction induced by α-Syn fibrils. Intracerebral injection of 24-OHC enhances the spread of α-Syn pathology and dopaminergic neurodegeneration via elevated X-box binding protein 1 (XBP1) and lymphocyte-activation gene 3 (LAG3) levels. Thus, elevated CYP46A1 and 24-OHC promote neurotoxicity and the spread of α-Syn via the XBP1–LAG3 axis. Strategies aimed at inhibiting the CYP46A1-24-OHC axis and LAG3 could hold promise as disease-modifying therapies for PD.

## Abstract 2

The cholesterol 24-hydroxylase (CYP46A1) has been linked to neurodegenerative disease, but it’s direct contribution to Parkinson’s disease is unknown. This study shows that dysregulation of cholesterol metabolism in the central nervous system plays a role, and that CYP46A1 activity promotes the formation of Lewy bodies and death of dopaminergic neurons.

## Introduction

Parkinson’s disease (PD) is the second most common neurodegenerative disease that leads to slowness of movement, tremor, rigidity, cognitive impairment, and neuropsychiatric symptoms [1]. Pathologically, PD is characterized by the accumulation of α-synuclein (α-Syn) in Lewy bodies/Lewy neurites (LBs/LNs) and the degeneration of dopaminergic neurons in the substantia nigra pars compacta (SNpc) [2–4]. The etiology of PD is incompletely understood but is believed to be multifactorial, involving both genetic and environmental factors [5]. α-Syn is a 140-amino acid cytoplasmic protein that is found mainly within presynaptic nerve terminals [6]. α-Syn remains unfolded in the cytosol or forms an α-helix structure in association with cell membranes. In the brains of patients with PD and other synucleinopathies, α-Syn undergoes polymerization into insoluble *β*-sheet-rich protein fibrils [7,8].

Converging lines of evidence indicate that α-Syn fibrils can spread in a prion-like manner in the brain, leading to self-propagation and cell-to-cell transmission of protein aggregates [9–11]. During the progression of PD, α-Syn pathology develops in a stereotypical pattern [12,13]. Single intrastriatal injection of α-Syn preformed fibrils (PFFs) triggers the aggregation of endogenous α-Syn and dopaminergic neurodegeneration in non-transgenic mice [11]. These studies indicate that α-Syn aggregation and cell-to-cell transmission are triggering events upstream of neuronal dysfunction and degeneration in PD. Lymphocyte-activation gene 3 (LAG3) was shown to mediate the cell-to-cell transmission of pathological α-Syn within the brain [14]. Although it is clear that α-Syn aggregation underlies the pathology of PD, what drives the spread of α-Syn remains unclear.

Recently, abnormal cholesterol metabolism in neurodegenerative diseases has been increasingly recognized [15–17]. Neurons utilize various mechanisms to manage excess cholesterol, including esterification into cholesterol esters stored within intracellular lipid droplets and their excretion via the ABCA1 transporter [18,19]. The principal route for eliminating surplus intracellular cholesterol is facilitated by cholesterol 24-hydroxylase (CYP46A1), which enzymatically converts cholesterol into 24S-hydroxycholesterol (24-OHC) [20]. 24-OHC and other oxysterols can permeate the blood–brain barrier (BBB) or transit to the bloodstream through the cerebrospinal fluid (CSF). Oxysterol homeostasis in the brain is meticulously regulated to maintain precise levels in each region [19]. The role of these cholesterol oxidation products in neurodegeneration remains unclear, necessitating further research to elucidate their precise function.

Interestingly, lipids and membranous organelles are the major constituents of LBs [21]. Various types of clinical evidence indicate that the levels of 24-OHC in the cerebral spinal fluid (CSF) are increased in PD patients and are correlated with the duration of the disease [20]. Moreover, studies have demonstrated a very significant association between SNPs in CYP46A1 and the occurrence of PD [22]. However, it remains unknown whether CYP46A1 and 24-OHC play vital roles in α-Syn pathology and the onset of PD. Herein, we evaluated the role of CYP46A1 and 24-OHC in α-Syn pathology. Strikingly, we found that 24-OHC exacerbates neurodegeneration in a mouse model injected with α-Syn fibrils, whereas genetic ablation of CYP46A1 is associated with less damage. Furthermore, 24-OHC promotes α-Syn aggregation both in vitro and in vivo. Compared with pure α-Syn fibrils, α-Syn fibrils formed in the presence of 24-OHC presented increased seeding activity and neurotoxicity. Moreover, 24-OHC increases the expression of LAG3 by activating transcription factor X-box binding protein 1 (XBP1) and promotes the cell-to-cell transmission of the pathologic α-Syn strain. These results suggest that CYP46A1 and 24-OHC play crucial roles in nigrostriatal dopaminergic degenerate on and α-Syn pathology.

## Results

### CYP46A1 and 24-OHC are increased in PD patients and PD model mice

**Fig 1:**
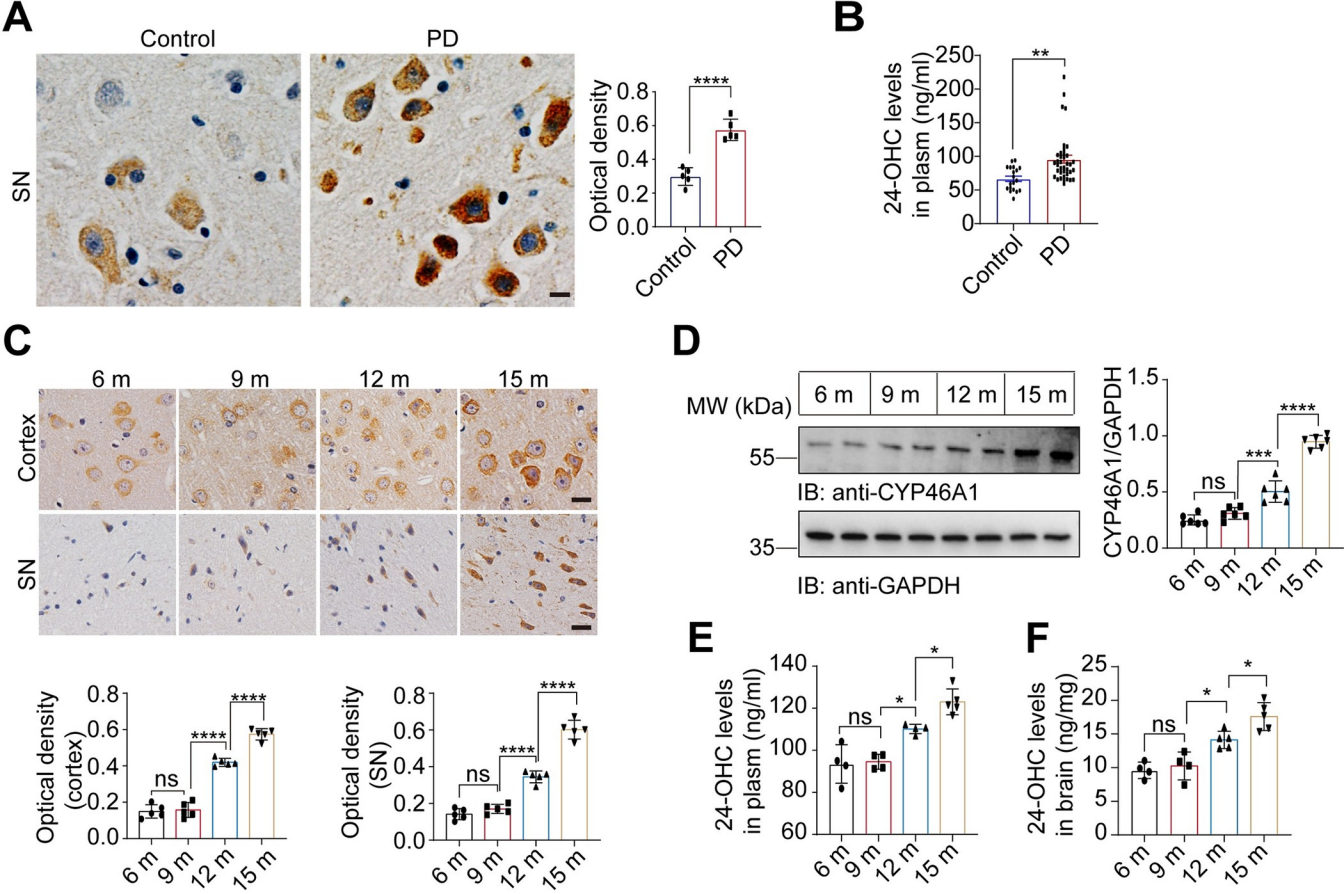
CYP46A1 and 24-OHC are up-regulated in PD patients and PD model mice. ** (A)** Immunohistochemistry showing the levels of CYP46A1 in the SNpc of PD patients (*n* = 5 subjects in each group). Scale bar, 20 μm. **(B)** Levels of 24-OHC in the plasma of PD patients (*n* = 38) and control subjects (*n* = 19) determined by LC–MS. Student’s *t* test with Welch’s correction. **P < 0.01. **(C)** Representative immunohistochemistry images of CYP46A1 in the cortex and SN of α-Syn A53T transgenic mice at different ages. Scale bar, 20 μm. Bar graphs are the optical density (*n* = 5 mice per group). **(D)** Immunoblotting showing CYP46A1 levels in the striatal lysates of α-Syn A53T transgenic mice at different ages. GAPDH was used as the loading control (*n* = 6 mice per group). **(E)** LC–MS analysis of plasma 24-OHC levels in α-Syn A53T transgenic mice at different ages (*n* = 4–5 mice per group). **(F)** LC–MS analysis of 24-OHC levels in the striatal lysates of α-Syn A53T transgenic mice at different ages (*n* = 4–5 mice per group). All data are means ± SEM. One-way ANOVA with Tukey’s multiple comparisons test. *P < 0.05, ****P < 0.001, ****P < 0.0001, and ns, not significant. Underlying data can be found in S1 Data. The uncropped blots are included in S1 Raw Images. PD, Parkinson’s disease; SN, substantia nigra; SNpc, substantia nigra pars compacta.

We first examined the levels of CYP46A1 in the postmortem substantia nigra (SN) samples from patients with PD. Compared with those in control subjects, the levels of CYP46A1 were increased in PD patients (**Fig 1A**). 24-OHC in CSF can enter the systemic circulation and be excreted in the urine, providing an indicator of brain cholesterol metabolism [19]. Consequently, plasma 24-OHC levels can serve as a proxy for brain 24-OHC levels in patients. LC–MS revealed that the levels of 24-OHC in the plasma of PD patients were greater than those in the plasma of age- and sex-matched control subjects (**Fig 1B and S1 Table**). Aging is the most important risk factor for PD. We found that the levels of CYP46A1 were elevated during aging in the brains of α-Syn A53T transgenic mice (line M83) (**Fig 1C and 1D**). The same results were found in the brains of aged wild-type mice **(S1 Fig)**. Furthermore, the levels of 24-OHC in the plasma and brain tissue of M83 mice also increased with age (**Fig 1E and 1F**). These results suggest that both CYP46A1 and 24-OHC increase in an age-dependent manner and are elevated in PD patients, PD model mice, and aged wild-type mice.

### α-Syn pathology and its spread are significantly reduced after CYP46A1 removal in vivo

**Fig 2:**
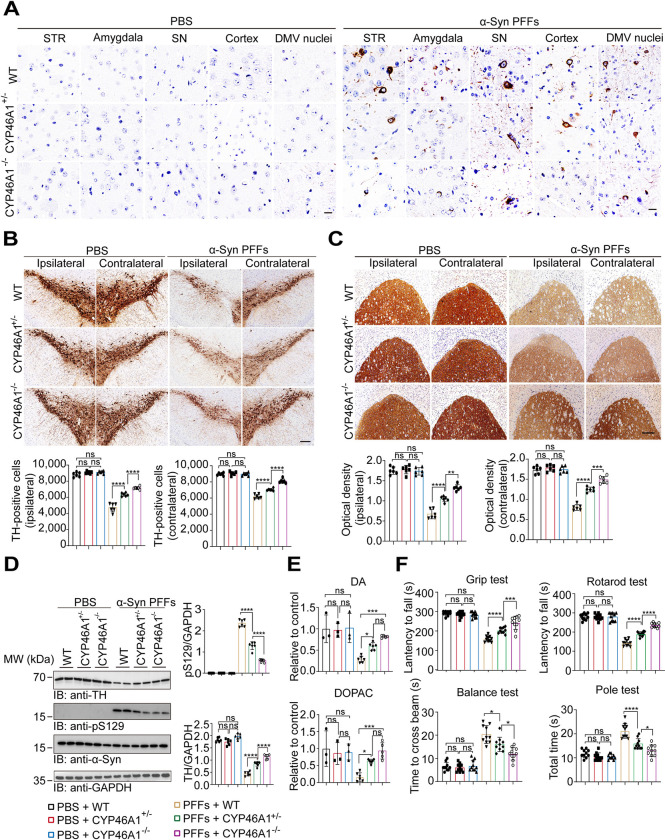
α-Syn pathology and its spread are significantly reduced after CYP46A1 removal in vivo. WT, CYP46A1^**+/−**^, and CYP46A1^−/−^ mice were injected with α-Syn PFFs (5 μg). **(A)** Ipsilateral pS129 immunohistochemistry of various brain regions at 180 dpi. Scale bar, 20 μm. STR: striatum. SN: substantia nigra. DMV: dorsal motor nucleus of the vagus nerve. **(B)** Representative images and quantification of TH-positive cells in the ipsilateral and contralateral SNpc. Scale bar, 100 μm. *n* = 6 mice per group. **(C)** Representative images and quantification of TH-positive cells in the ipsilateral and contralateral striatum. Scale bar, 100 μm. *n* = 6 mice per group. **(D)** Immunoblots and quantification of pS129 and TH in the ipsilateral striatum. *n* = 6 mice per group. **(E)** The contents of striatal DA and DOPAC in the ipsilateral striatum were measured by HPLC. *n* = 3–6 mice per group. **(F)** Behavioral tests, including the rotarod test, wire hang test, pole test, and balance beam test. *n* = 10 mice per group. All data are means ± SEM. One-way ANOVA with Tukey’s multiple comparisons test. *P < 0.05, **P < 0.01, ***P < 0.001, ****P < 0.001. ns, not significant. Underlying data can be found in S1 Data. The uncropped blots are included in S1 Raw Images. dpi, days postinjection; HPLC, high-performance liquid chromatography; PFF, preformed fibril; SNpc, substantia nigra pars compacta; WT, wild type.

α-Syn pathology is an important pathological feature of PD [2–4]. To verify the role of CYP46A1 in the propagation of α-Syn pathology in vivo, we injected α-Syn PFFs into the right striatum of wild-type (WT) mice, CYP46A1 half knockout (CYP46A1^+/−^) mice, and CYP46A1 full knockout (CYP46A1^−/−^) mice. The expression of CYP46A1 in the brain was confirmed by western blotting (**S2A Fig**). As expected, depletion of the CYP46A1 gene dramatically reduced the levels of 24-OHC in both plasma and brain tissue (**S2B and S2C Fig**). The levels of cholesterol and other byproducts in the CYP46A1 knockout mice were also detected (**S3A and S3B Fig**). The extent of α-syn pathology and dopaminergic neurodegeneration was determined at 180 days postinjection (dpi). Immunostaining revealed that the density of pS129-positive α-Syn inclusions decreased in CYP46A1 knockout mice in a gene dose-dependent manner (**Figs 2A and S4**). Intrastriatal injection of α-Syn PFFs led to an approximate 50% loss of DA neurons in the SNpc of WT mice. In contrast, the loss of dopaminergic neurons in the SNpc and the loss of dopaminergic terminals were strongly attenuated in the CYP46A1^+/−^ and CYP46A1^−/−^ mice (**Fig 2B and 2C**). Western blot analysis confirmed that genetic ablation of CYP46A1 alleviated pS129 accumulation and loss of TH in the striatum (**Fig 2D**). High-performance liquid chromatography (HPLC) revealed that the decrease in striatal dopamine and DOPAC was attenuated in the CYP46A1^+/−^ and CYP46A1^−/−^ mice compared with the WT mice (**Fig 2E**). Moreover, CYP46A1 knockdown alleviated these behavioral impairments (**Fig 2F**). Thus, CYP46A1 knockdown relieves the spread of α-Syn pathology and the loss of dopaminergic neurons.

### Elevated CYP46A1 promotes α-Syn aggregation by 24-OHC in vitro

**Fig 3:**
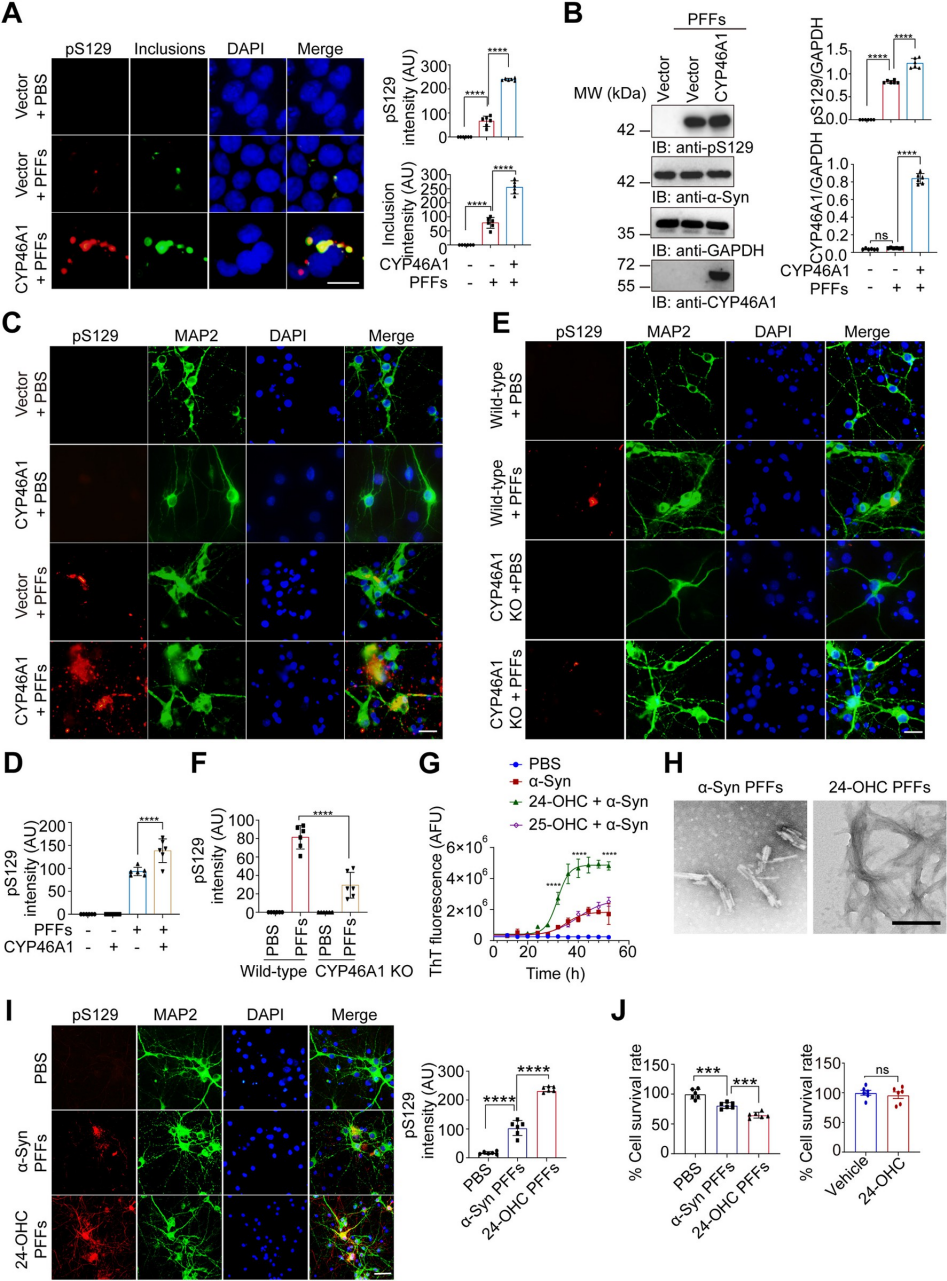
Elevated CYP46A1 promotes α-Syn aggregation by 24-OHC in vitro. **(A, B)** α-Syn-GFP HEK293 cells were transfected with His-CYP46A1 and then treated with α-Syn PFFs for 48 h. PBS was used as the transduction control for PFFs, while the His vector was used as a transfection control for His-CYP46A1. **(A)** Triton X-100 (1%) in PBS was used to permeabilize soluble proteins to observe insoluble α-Syn inclusions. Images of pS129 (red) and insoluble α-Syn inclusions (green). *n* = 6 independent experiments. AU, arbitrary units. **(B)** Western blot analysis of α-Syn, CYP46A1, and pS129. Scale bar, 20 μm. *n* = 6 independent experiments. **(C, D)** Primary neurons were infected with AAV-CYP46A1 or control AAVs and then treated with α-Syn PFFs for 9 days. pS129 (red) and MAP 2 (green) staining. Quantitative analysis of pS129. *n* = 6 independent experiments. **(E, F)** Primary neurons from WT or CYP46A1 KO mice were transduced with α-Syn PFFs for 9 days. Shown are MAP2 (green) and pS129 (red). Quantitative analysis of pS129. *n* = 6 independent experiments. **(G)** Kinetics of α-Syn fibrillation (2 mg/ml monomer) in the presence or absence of 30 μm 24-OHC or 25-OHC in the real-time ThT fluorescence assay (*n* = 6 independent experiments). AFU, arbitrary fluorescence unit. **(H)** Ultrastructural images of α-Syn PFFs and 24-OHC PFFs revealed by TEM. Scale bars, 200 nm. **(I, J)** Primary neurons were treated with α-Syn PFFs and 24-OHC PFFs for 9 days. **(I)** Double immunofluorescence of pS129 (red) and MAP2 (green) in primary neuron. Scale bar, 20 μm. **(J)** Cell viability of neurons was detected by Cell Counting Kit-8 (CCK8) after treatment with 24-OHC or 24-OHC PFFs (*n* = 6 independent experiments). All data are means ± SEM. One-way ANOVA with Tukey’s multiple comparisons test. ****P < 0.0001. ns, not significant. Underlying data can be found in S1 Data. The uncropped blots are included in S1 Raw Images. PFF, preformed fibril; TEM, transmission electron microscopy; WT, wild type.

The abnormal aggregation of α-Syn is a key characteristic of α-Syn pathology [9–11]. To investigate the effect of CYP46A1 on α-Syn aggregation, we used HEK293 cells stably transfected with α-Syn-GFP (α-Syn-GFP HEK293 cells) as reporter cells. To determine the effect of CYP46A1 on α-syn aggregation, α-Syn-GFP HEK293 cells were transfected with his-CYP46A1 and then treated with α-Syn PFFs for 48 h. Overexpression of CYP46A1 increased the aggregation and phosphorylation of α-Syn (pS129), which is characteristic of LBs (**Figs 3A, 3B, and S5**). A similar effect was found in cultured primary neurons overexpressing CYP46A1 (**Fig 3C and 3D**). When cortical neurons isolated from CYP46A1-knockdown mice or WT mice were treated with α-syn PFFs for 9 days, the pS129 level was greater in CYP46A1-knockdown neurons than in WT neurons (**Fig 3E and 3F**). To explore whether CYP46A1 directly interacts with α-Syn to promote α-Syn aggregation, HEK293 cells were simultaneously transfected with GST-α-Syn and CYP46A1, followed by a GST pull-down assay. Tau, which has been shown to directly interact with α-Syn [23], was used as a positive control. The results showed that CYP46A1 does not interact with α-Syn (**S6 Fig**).

Although CYP46A1 promotes α-Syn aggregation, it does not directly interact with α-Syn. Instead, its major product, 24-OHC, is a significant candidate for promoting α-Syn aggregation. To investigate the effect of 24-OHC on α-Syn aggregation, we conducted a real-time thioflavin T (ThT) fluorescence assay to monitor the kinetics of α-Syn fibrillation. Fibrils were generated by shaking monomeric α-Syn in the presence or absence of 30 μm 24-OHC for 7 days at 37°C. The concentration of 24-OHC is based on the levels reported in previous literature, which reported that the concentration of 24-OHC in brain tissue was 8.6 to 15.1 ng/mg net weight (equal to approximately 30 μm) [24]. The aggregation of α-Syn was dramatically accelerated in the presence of 24-OHC. Conversely, 25-hydroxycholesterol (25-OHC), another oxidized cholesterol derivative, did not influence α-Syn aggregation (**Fig 3G**). The α-Syn fibers formed in the presence of 24-OHC were termed 24-OHC PFFs. Transmission electron microscopy (TEM) revealed that 24-OHC PFFs were coarser, longer, and more compact than pure α-Syn PFFs were **(Fig 3H)**. These findings strongly suggest that elevated CYP46A1 promotes abnormal α-Syn aggregation through the action of 24-OHC.

### 24-OHC accelerates the seeding activity and neurotoxicity of α-Syn fibrils in vitro

Converging lines of evidence indicate that α-Syn fibrils can propagate in a prion-like manner, inducing the aggregation of normal proteins [25]. To further investigate the effect of 24-OHC on the seeding activity of α-Syn fibrils in cells, we transduced α-Syn-HEK293 cells with either α-Syn PFFs or 24-OHC PFFs. This transduction induced the formation of insoluble α-Syn inclusions. After permeabilization with 1% TX-100 to remove soluble α-Syn, a greater number of insoluble inclusions were observed in the 24-OHC PFF-treated cells. These inclusions were positive for pS129 **(S7A Fig)**. Immunoblotting further confirmed that the levels of pS129 were elevated in 24-OHC PFF-treated cells **(S7B Fig)**. Additionally, 24-OHC PFFs presented greater seeding activity than did α-Syn PFFs in primary neurons **(Fig 3I)**. Compared with α-Syn PFFs, 24-OHC PFFs also reduced the survival of cultured neurons **(Fig 3J)**. Thus, 24-OHC not only promotes the aggregation of α-Syn but also results in the formation of α-Syn fibrils with increased seeding activity and neurotoxicity.

### The seeding activity of 24-OHC PFFs is greater than that of α-Syn PFFs in vivo

**Fig 4:**
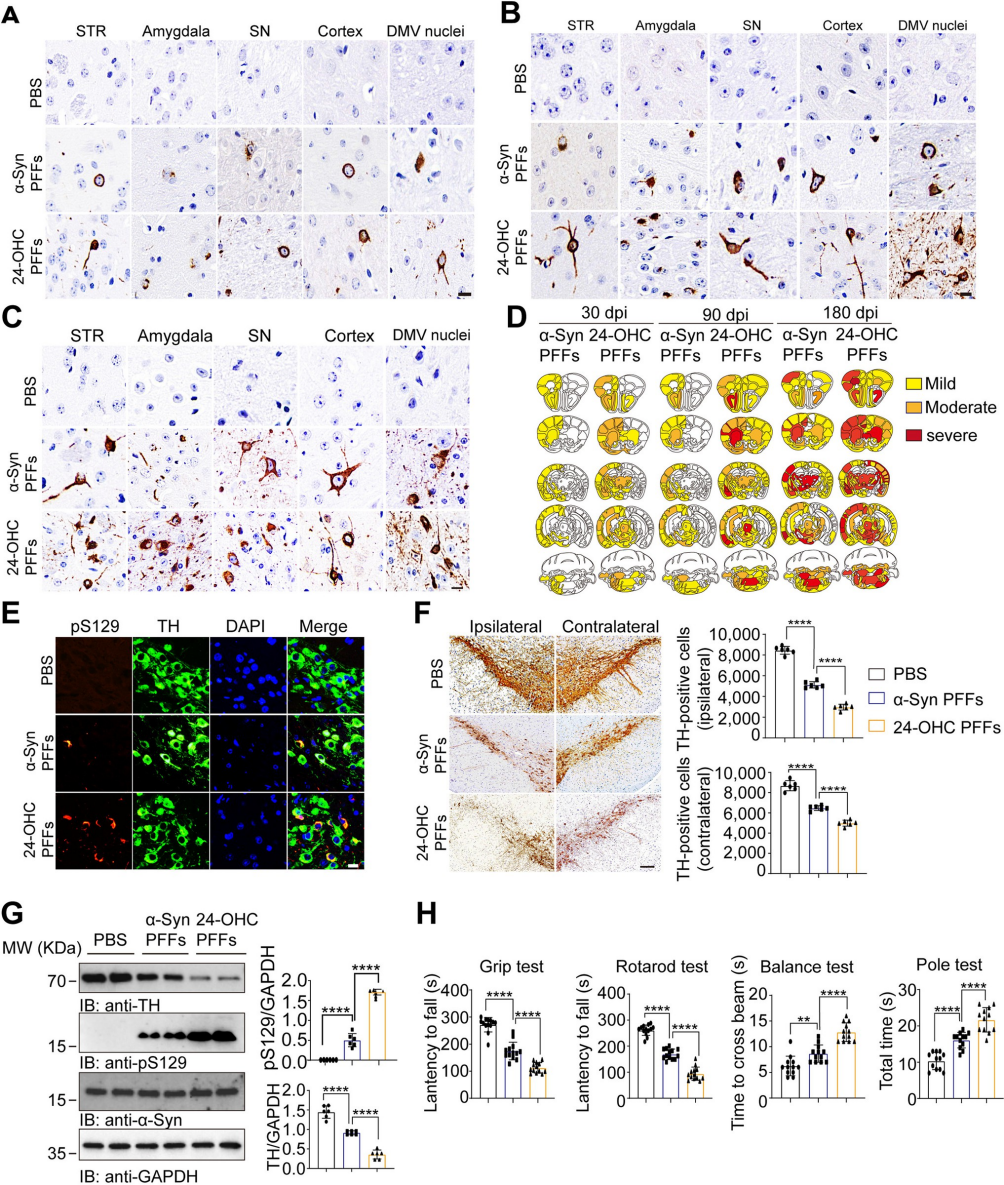
The seeding activity of 24-OHC PFFs was greater than that of α-Syn PFFs in vivo. Three-month-old WT mice received a single intrastriatal injection of α-Syn PFFs or 24-OHC PFFs (5 μg). **(A)** Ipsilateral pS129 immunohistochemistry of various brain regions at 30 dpi. Scale bar, 20 μm. STR: striatum. SN: substantia nigra. DMV: dorsal motor nucleus of the vagus nerve. **(B)** Ipsilateral pS129 immunohistochemistry of various brain regions at 90 dpi. Scale bar, 20 μm. **(C)** Ipsilateral pS129 immunohistochemistry of various brain regions at 180 dpi. Scale bar, 20 μm. **(D)** Heatmap of pS129 pathology in mice at 30, 90, and 180 dpi. **(E)** Co-localization of pS129 (red) and TH (green) in the SNpc at 180 dpi. Scale bar, 20 μm. **(F)** Representative immunohistochemistry images and quantification of TH-positive neurons in the ipsilateral and contralateral SNpc at 180 dpi. Scale bar, 100 μm. *n* = 6 mice per group. **(G)** Immunoblots and quantification of pS129 and TH in the ipsilateral striatum at 180 dpi. *n* = 6 mice per group. **(H)** Behavioral tests, including the balance test, grip test, pole test, and rotarod test, were performed at 180 dpi. *n* = 12 mice per group. All data are means ± SEM. One-way ANOVA with Tukey’s multiple comparisons test (*n* = 6 independent experiments). ****P < 0.0001. Underlying data can be found in S1 Data. The uncropped blots are included in S1 Raw Images. dpi, days postinjection; PFF, preformed fibril; SNpc, substantia nigra pars compacta; WT, wild type.

To investigate the effect of 24-OHC on the toxicity of α-Syn fibrils, we injected equal amounts of α-Syn PFFs and 24-OHC PFFs into the unilateral striatum of WT mice and monitored α-Syn pathology at 30, 90, and 180 dpi. Deposits of pS129 were visible at the injection site at 30 dpi. The extent of pS129-positive LB-like inclusions was greater in the mice injected with 24-OHC PFFs than in the mice injected with α-Syn PFFs **(Fig 4A)**. α-Syn pathology spreads in the mouse brain at 90 and 180 dpi. More pS129-positive LB-like α-Syn aggregates were found in the mice injected with 24-OHC PFFs than in the mice injected with α-Syn PFFs **(Fig 4B and 4C)**. Mapping of pS129 pathology in mice at 30, 90, and 180 dpi revealed time-dependent dissemination of α-Syn pathology between 30 and 180 dpi **(Fig 4D)**.

### 24-OHC PFFs induce more severe nigrostriatal dopaminergic degeneration and motor impairments than α-Syn PFFs

To compare the effects of α-Syn PFFs and 24-OHC PFFs on the degeneration of the nigrostriatal dopaminergic pathway, we stained the SNpc with TH. Stronger pS129 signals were observed in TH-positive neurons in the SNpc of mice injected with 24-OHC PFFs than in those injected with α-Syn PFFs **(Fig 4E)**. Compared with that in α-Syn PFF-injected mice, the number of TH-positive neurons in the SNpc ipsilateral to the injection site was significantly lower in mice injected with 24-OHC PFFs **(Fig 4F)**. Western blot analysis further confirmed lower levels of TH and higher levels of pS129 in the striatum of mice injected with 24-OHC PFFs than in those injected with α-Syn PFFs **(Fig 4G)**. These findings suggest that 24-OHC PFFs induce more severe degeneration of the nigrostriatal dopaminergic pathway than α-Syn PFFs.

Furthermore, we evaluated the motor function of mice injected with α-Syn PFFs and 24-OHC PFFs using the grip test, rotarod test, balance test, and pole test. At 180 dpi, the motor impairment of the mice injected with 24-OHC PFFs was more pronounced than that of the mice injected with α-Syn PFFs **(Fig 4H)**. Thus, 24-OHC PFFs induce more severe α-Syn pathology, loss of dopaminergic neurons, and PD-like motor impairments.

### Exogenous 24-OHC facilitates the propagation of α-Syn pathology

**Fig 5:**
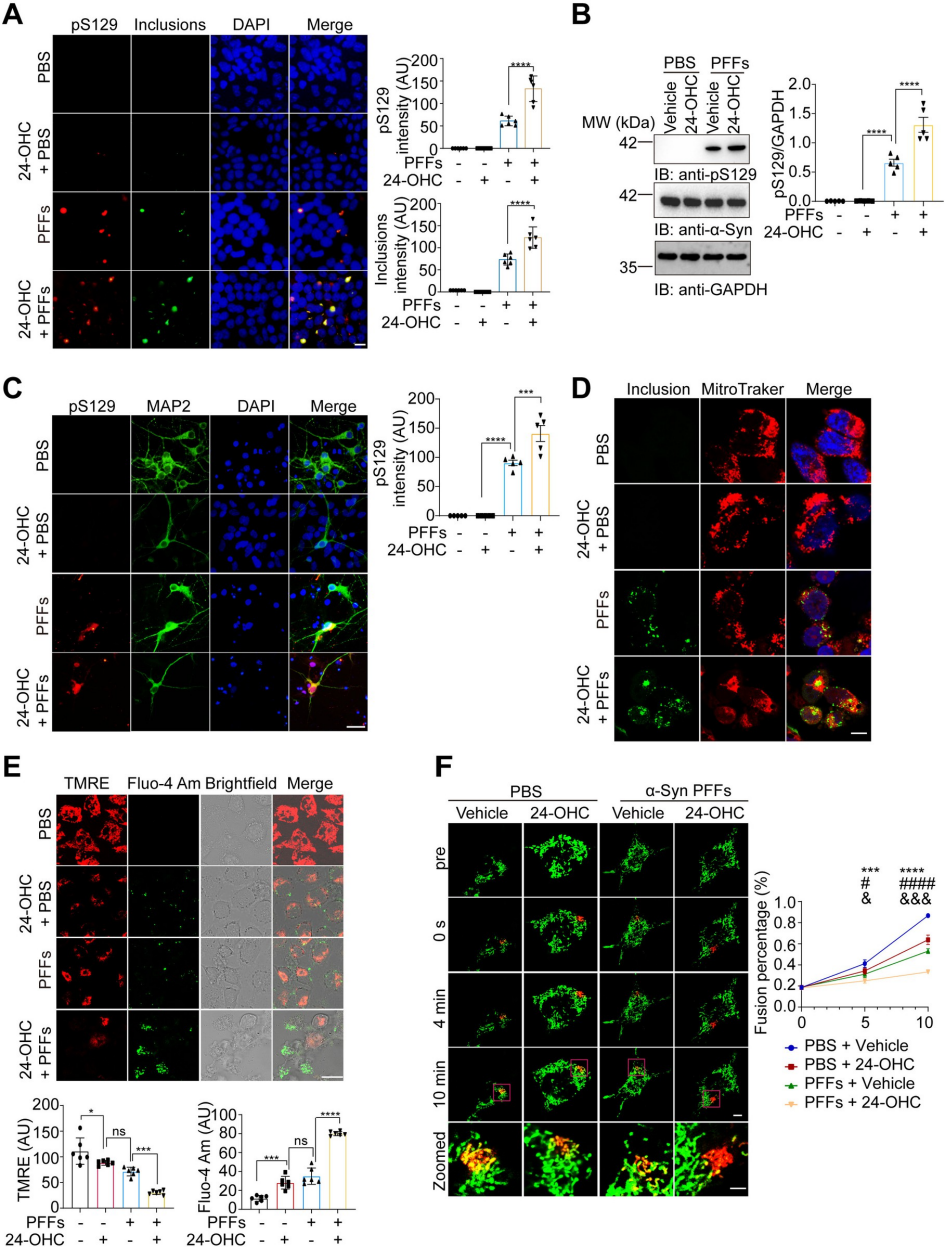
24-OHC facilitates the propagation of α-Syn pathology and mitochondrial dysfunction. **(A, B)** α-Syn-GFP HEK293 cells were transduced with α-Syn PFFs in the presence or absence of 24-OHC (30 μm) for 48 h. **(A)** Triton X-100 (1%) in PBS was used to permeabilize soluble proteins to observe insoluble α-Syn inclusions. Shown are the representative immunostaining and quantification of the insoluble α-Syn inclusions (green) and pS129 (red). AU, arbitrary units. *n* = 6 independent experiments. **(B)** Immunoblots of phosphorylated α-Syn (pS129) and α-Syn. *n* = 5 independent experiments. **(C)** Neurons were exposed to α-Syn PFFs in the presence or absence of 24-OHC (30 μm) for 9 days. Shown are pS129 (red) and MAP2 (green). AU, arbitrary unit. *n* = 5 independent experiments. **(D)** α-Syn-GFP HEK293 cells were transduced with α-Syn PFFs in the presence or absence of 24-OHC (30 μm) for 48 h. Representative images showing the co-localization of mitochondria (MitoTracker, red) and α-Syn inclusions (green). Scale bar, 8 μm. *n* = 6 independent experiments. **(E)** SH-SY5Y cells were transduced with α-Syn PFFs in the presence or absence of 24-OHC for 48 h. TMRM staining of the mitochondrial membrane potential (red) and Fluo-4 Am (green) intracellular calcium concentration. Scale bar, 20 μm. *n* = 6 independent experiments. All data are means ± SEM. One-way ANOVA with Tukey’s multiple comparisons test. *P < 0.05, **P < 0.01, ***P < 0.001, ****P < 0.001, and ns, not significant. AU, arbitrary unit. **(F)** SH-SY5Y cells were transfected with Mitodendra2 and then transduced with α-Syn PFFs in the presence or absence of 24-OHC (30 μm) for 48 h. Cells were live-imagined using a confocal microscope. A subpopulation of mitochondria within a single cell was subjected to full-photon shifting by exposing the region of interest to 405-nm fluorescence (10% power) for 2 s. Representative images showing mitochondrial fusion. Red: shifted mitochondria; Green: original mitochondria; Yellow: fused mitochondria. Scale bar, 8 μm. The bar graph shows the quantification of mitochondrial fusion. One-way ANOVA with Tukey’s multiple comparisons test. *n* = 6 independent experiments. PFF + 24-OHC group vs. PFF + vehicle group: ^&^*P* < 0.05 and ^&&&^*P* < 0.001. PFFs + 24-OHC group vs. PBS + vehicle group: ***P < 0.001 and ****P < 0.0001. PFFs + vehicle group vs. PBS + vehicle group: ^#^*P* < 0.05 and ^####^*P* < 0.05. Underlying data can be found in S1 Data. The uncropped blots are included in S1 Raw Images. PFF, preformed fibril.

In addition to abnormal aggregation, the propagation of α-Syn fibrils represents another significant feature of α-Syn pathology [25]. To further investigate the impact of exogenous 24-OHC on the propagation of α-Syn fibrils in vitro, α-Syn-GFP HEK293 cells were transduced with α-Syn PFFs together with 24-OHC or vehicle. The concentrations used were selected according to previous studies [26]. After transduction for 48 h, soluble α-Syn was eliminated via the addition of 1% TX-100. More insoluble pS129-positive α-Syn aggregates were found in the presence of 24-OHC. 24-OHC alone did not trigger α-Syn aggregation in the absence of exogenous α-Syn PFFs (**Fig 5A**). Western blot analysis revealed that the levels of pS129 were elevated in cells transduced with α-Syn PFFs, which was enhanced by 24-OHC (**Fig 5B**). Furthermore, 24-OHC facilitated the phosphorylation and aggregation of endogenous α-Syn induced by α-Syn PFFs in primary cultured neurons (**Fig 5C**). Thus, 24-OHC facilitates the pathology induced by α-Syn PFFs in vitro.

### 24-OHC exacerbates the mitochondrial dysfunction induced by α-Syn PFFs

Mitochondrial dysfunction plays an important role in the pathogenesis of PD. To determine the subcellular localization of insoluble α-Syn inclusions, α-Syn-GFP HEK293 cells were exposed to α-Syn PFFs in the presence or absence of 24-OHC for 48 h. Immunofluorescence staining revealed that α-Syn inclusions colocalized with mitochondria. 24-OHC dramatically enhanced the co-localization of α-Syn inclusions with mitochondria (**Fig 5D**). We further tested the effects of 24-OHC on the mitochondrial membrane potential and intracellular Ca^2+^ concentration in SH-SY5Y cells via TMRE and Fluo-4 Am, respectively. α-Syn PFFs decreased the mitochondrial membrane potential and increased the intracellular Ca^2+^ concentration, which was exacerbated by 24-OHC (**Fig 5E**). Furthermore, we investigated mitochondrial fusion activity in SH-SY5Y cells and found that mitochondrial fusion dynamics were impaired in the presence of 24-OHC (**Fig 5F**). These results suggest that 24-OHC exacerbates the mitochondrial dysfunction induced by α-Syn PFFs.

### Intracerebroventricular injection of 24-OHC aggravates neurodegeneration in vivo

**Fig 6:**
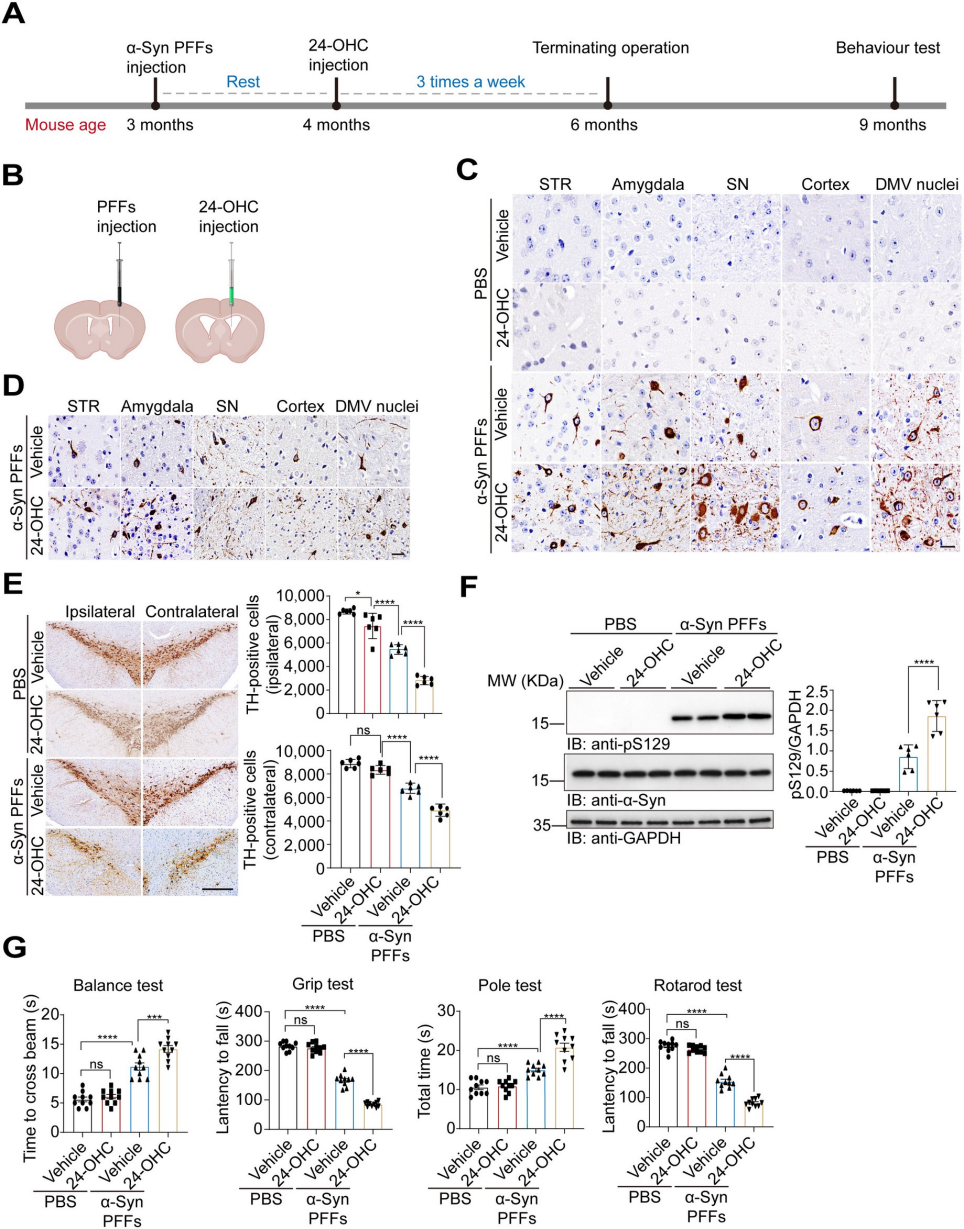
Intracerebroventricular injection of 24-OHC aggravates neurodegeneration. **(A)** Schematic representation of the mouse experiments. **(B)** Schematic of the injection site. The mice were injected with α-Syn PFFs and then intracerebroventricularly injected with 24-OHC (100 μg/kg body weight). **(C)** Ipsilateral pS129 in various brain regions. Scale bar, 20 μm. STR: striatum. SN: substantia nigra. DMV: dorsal motor nucleus of the vagus nerve. **(D)** Representative immunohistochemistry images showing ipsilateral pS129-positive LB-like accumulation after PK digestion. Scale bar, 20 μm. **(E)** Representative immunohistochemistry and quantification of TH-positive cells in the ipsilateral and contralateral SNpc. Scale bar, 100 μm. *n* = 6 mice per group. **(F)** Immunoblots and quantification of pS129 in the ipsilateral striatum. *n* = 6 mice per group. **(G)** Behavioral tests, including the rotarod test, wire hang test, pole test, and balance beam test. *n* = 10 mice per group. All data are means ± SEM. One-way ANOVA with Tukey’s multiple comparisons test. *P < 0.05, **P < 0.01, ***P < 0.001, ****P < 0.001, and ns, not significant. Underlying data can be found in S1 Data. The uncropped blots are included in S1 Raw Images. PFF, preformed fibril; SNpc, substantia nigra pars compacta.

Since we found that 24-OHC intensifies α-Syn spreading and toxicity in vitro, we sought to determine whether 24-OHC regulates α-Syn pathology in vivo. Five micrograms of α-Syn PFFs were injected into the right striatum of 3-month-old mice. One month later, 24-OHC or vehicle was injected into the lateral ventricle 3 times a week for 8 weeks (**Fig 6A and 6B**). Immunohistochemistry revealed intraneuronal pS129-positive inclusions in brain regions, including the amygdala, SN, striatum, cortex, and dorsal motor nucleus of the vagus (DMV) nuclei. The extent of α-Syn pathology was more severe in the mice injected with 24-OHC (**Figs 6C and S8**). PK digestion assays revealed that the inclusions in the 24-OHC group were more resistant to PK than those in the vehicle group (**Fig 6D**). Furthermore, the number of dopaminergic neurons in the SNpc was decreased in mice injected with α-Syn PFFs and aggravated by 24-OHC injection (**Fig 6E**). Immunoblotting analysis confirmed increased pS129 levels in the striatum of mice injected with 24-OHC (**Fig 6F**). Moreover, 24-OHC injection promoted the behavioral impairments induced by PFFs (**Fig 6G**). Thus, 24-OHC promotes α-Syn pathology, the loss of dopaminergic neurons, and behavioral impairments.

### 24-OHC activates the XBP1/LAG3 axis in vivo and in vitro

**Fig 7:**
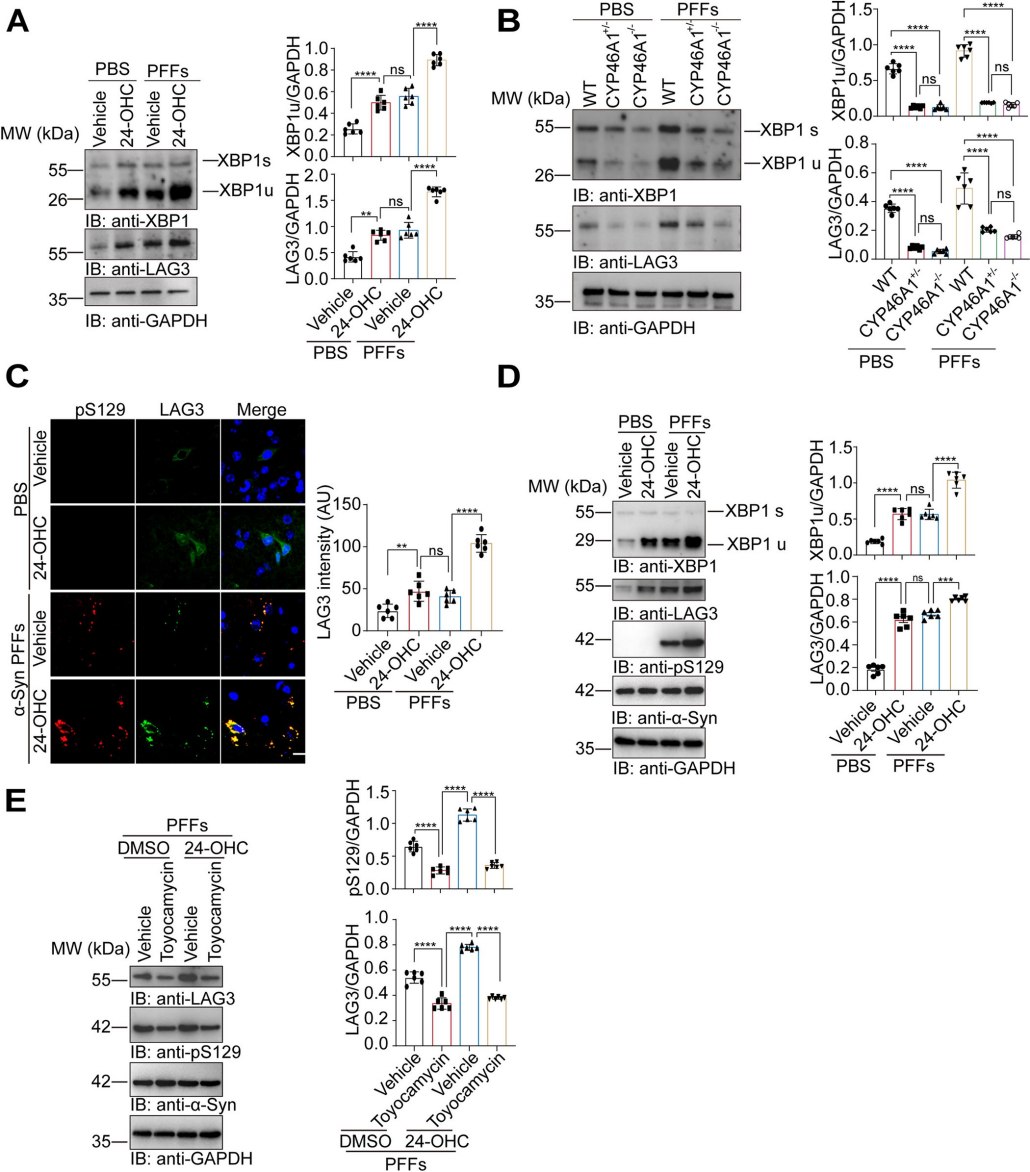
24-OHC activates the XBP1/LAG3 axis both in vivo and in vitro. **(A)** Immunoblots and quantification of XBP1 and LAG3 in the striatal tissues of WT mice injected with 24-OHC (100 μg/kg body weight) and α-Syn PFFs at 180 dpi (as in Fig 6). *n* = 6 mice per group. **(B)** Immunoblots and quantification of XBP1 and LAG3 in the striatum of CYP46A1 knockout mice injected with α-Syn PFFs at 180 dpi (as in Fig 2). *n* = 6 mice per group. **(C)** Representative images showing LAG3 (green) and pS129 (red) in the substantial nigra of mice injected with 24-OHC and PFFs (as in Fig 6). *n* = 6 mice per group. Scale bar, 20 μm. AU, arbitrary unit. **(D)** Immunoblots and quantification of LAG3 and XBP1 in α-Syn-GFP HEK293 cells transduced with α-Syn PFFs in the presence of 24-OHC (30 μm) for 48 h. *n* = 6 independent experiments. **(E)** Effect of the XBP1 inhibitor toyocamycin on the expression of LAG3 in α-Syn-GFP HEK293 cells treated with α-Syn PFFs and 24-OHC (30 μm). *n* = 6 independent experiments. **P < 0.01, ****P < 0.0001, and ns, not significant. Underlying data can be found in S1 Data. The uncropped blots are included in S1 Raw Images. dpi, days postinjection; LAG3, lymphocyte-activation gene 3; PFF, preformed fibril; WT, wild type; XBP1, X-box binding protein 1.

Cell-to-cell transmission mediates α-Syn pathology. It has been reported that LAG3 is involved in the intercellular transmission of α-Syn fibers [14]. Moreover, cholesterol promotes the expression of LAG3 by activating the transcription factor XBP1 [27]. Thus, we detected the levels of XBP1 and LAG3 in mice injected with 24-OHC. The results showed that 24-OHC treatment increased the expression of XBP1 and LAG3 in vivo (**Fig 7A**). XBP1 and LAG3 were decreased after genetic ablation of CYP46A1 in vivo (**Fig 7B**). Moreover, immunofluorescence revealed that LAG3 colocalized with pS129 in the mice injected with 24-OHC and PFFs (**Fig 7C**). We tested the effects of 24-OHC on the expression of LAG3 and XBP1 in α-Syn-GFP HEK293 cells. Interestingly, the levels of both LAG3 and XBP1 increased in the presence of 24-OHC (**Fig 7D**). To clarify the role of XBP1 in 24-OHC-induced LAG3 expression, toyocamycin, an XBP1 inhibitor, was used. The cells were exposed to 24-OHC in the presence or absence of 40 nM toyocamycin. The results showed that toyocamycin attenuated LAG3 expression induced by 24-OHC (**Fig 7E**), suggesting that XBP1 is responsible for the overexpression of LAG3 induced by 24-OHC.

### Activated XBP1 and LAG3 axis by 24-OHC enhances cell-to-cell transmission of α-Syn

**Fig 8:**
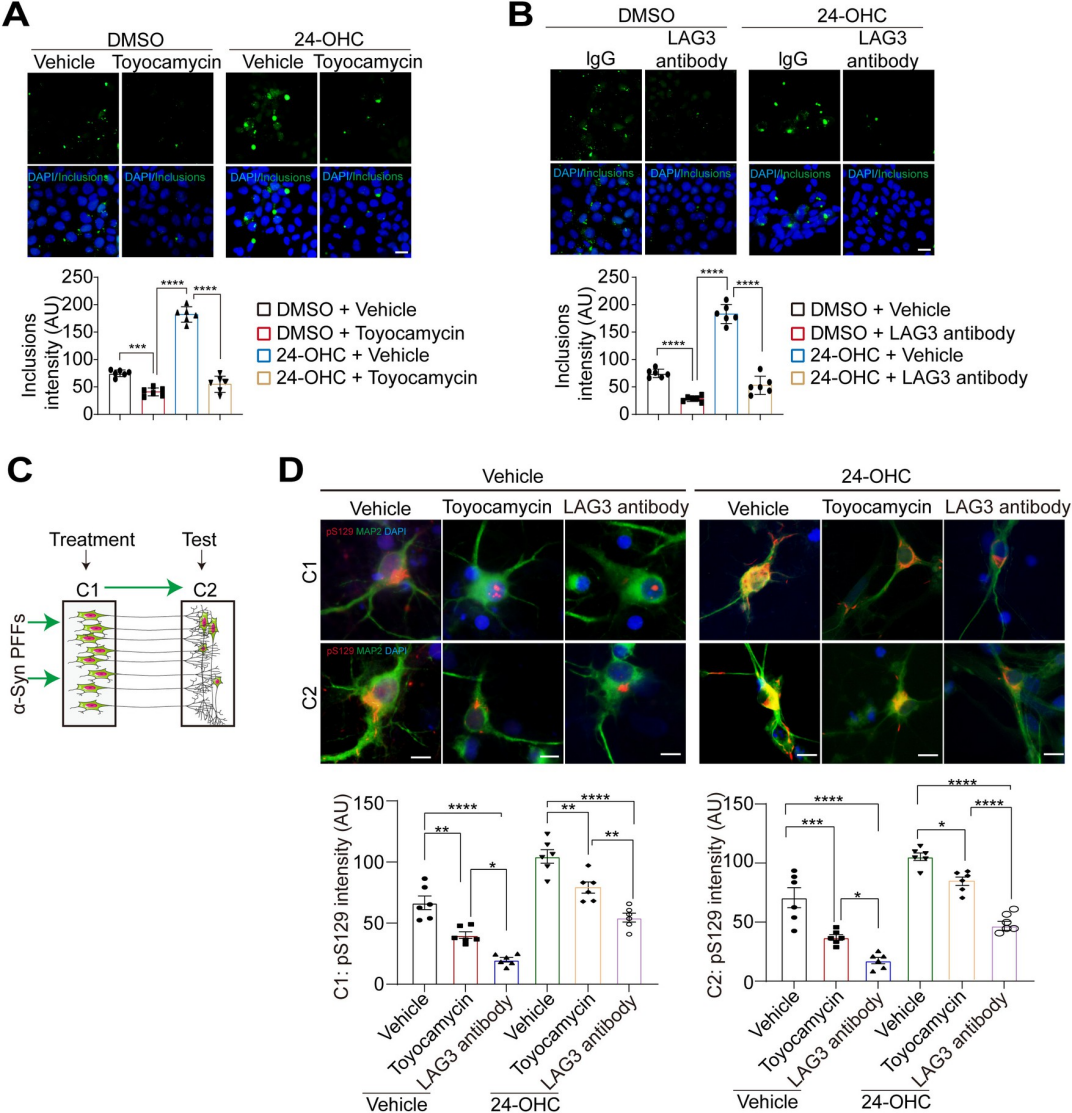
XBP1 and LAG3 mediate the effect of 24-OHC on the cell-to-cell transmission of α-syn. **(A)** Representative images of insoluble inclusions and pS129 in α-Syn-GFP HEK293 cells transduced with α-Syn PFFs in the presence or absence of 24-OHC (30 μm) and toyocamycin. *n* = 6 independent experiments. **(B)** Representative images of insoluble inclusions and pS129 in α-Syn-GFP HEK293 cells transduced with α-Syn PFFs in the presence of LAG3 antibody. *n* = 6 independent experiments. **(C)** Schematic representation of chambers in which neurons were cultured in chamber 1 (C1) and chamber 2 (C2). **(D)** Transmission of pathologic p129 from C1 to C2 at 14 days after treatment with α-syn PFFs and 24-OHC in combination with toyocamycin or an anti-LAG3 antibody. *n* = 6 independent experiments. All data are means ± SEM. One-way ANOVA with Tukey’s multiple comparisons test. *n* = 6 independent experiments. ns, not significant, *P < 0.05, *P < 0.01 ***P < 0.001, and ****P < 0.0001. Scale bar, 20 μm. AU, arbitrary unit. Underlying data can be found in S1 Data. LAG3, lymphocyte-activation gene 3; PFF, preformed fibril; XBP1, X-box binding protein 1.

**Fig 9:**
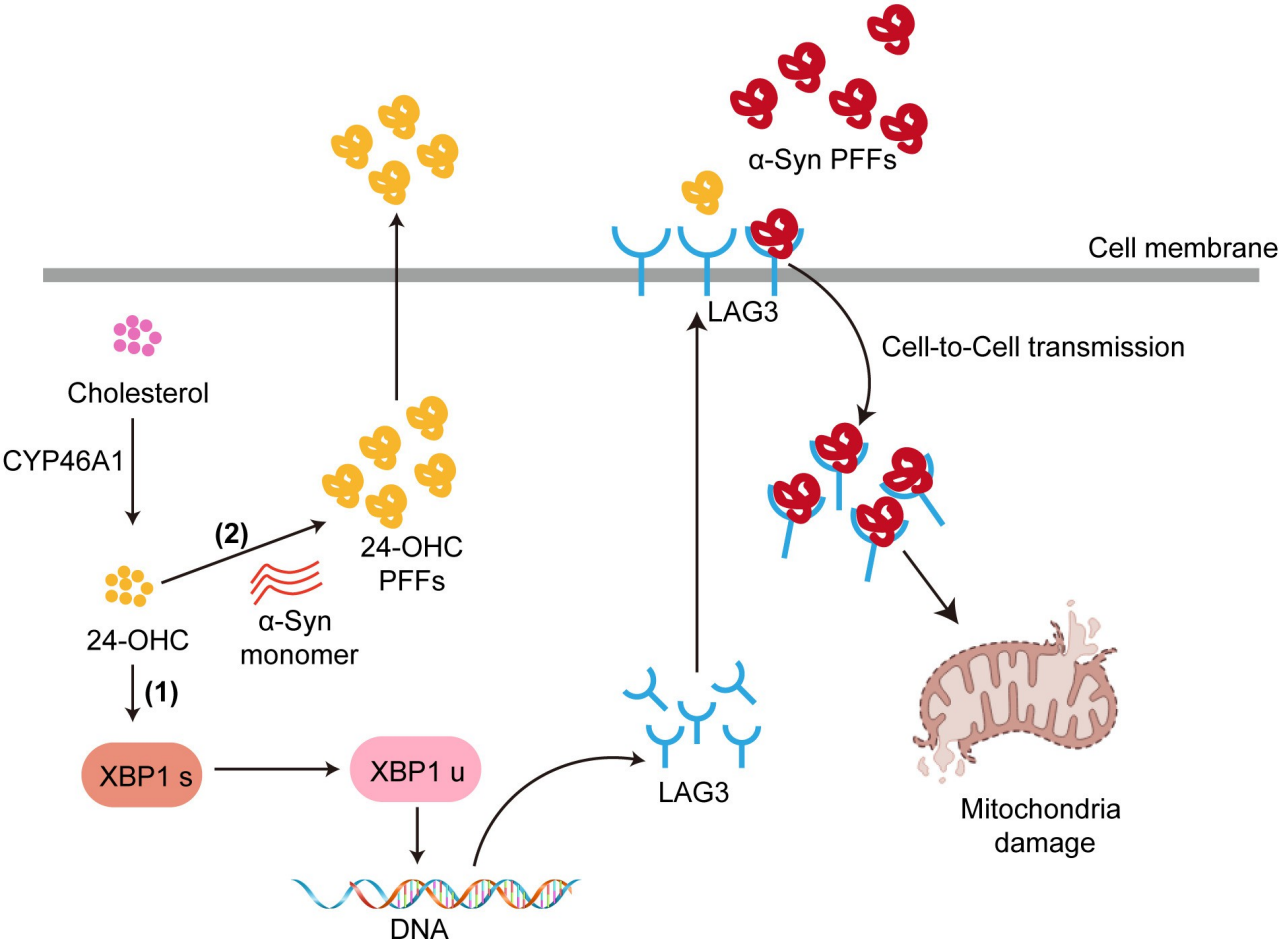
Schematic representation of CYP46A1 and 24-OHC in α-Syn pathology. During aging, overactivated CYP461 catalyzes cholesterol to produce 24-OHC, which promotes XBP1s splicing to produce the XBP1u fragment. XBP1u is incorporated into the nucleus and increases LAG3 expression. The membrane protein LAG3 binds to α-Syn PFFs and promotes the cell-to-cell transmission of α-Syn seeds. In addition, 24-OHC facilitates α-Syn assembly, resulting in the formation of morphologically different 24-OHC PFFs, which are more neurotoxic than pure α-Syn PFFs. LAG3, lymphocyte-activation gene 3; PFF, preformed fibril; XBP1, X-box binding protein 1.

To confirm the role of LAG3 and XBP1 in the cell-to-cell transmission of α-Syn, an LAG3 antibody was added to block LAG3 function. The seeding of α-Syn was dramatically attenuated in the presence of the anti-LAG3 antibody. Moreover, toyocamycin treatment prevented the formation of insoluble aggregates induced by α-Syn PFFs (**Fig 8A and 8B**). To examine the transmission of α-Syn PFFs and to establish the role of 24-OHC in the interneuron transmission of α-Syn, we used a microfluidic neuronal culture device with 2 chambers connected in tandem by a series of microgrooves separating the chambers (RD150, Xona Microfluidics, LLC). The medium volume in chamber 1 (C1) was 50 μl lower than that in chamber 2 (C2) to prevent diffusion of α-Syn PFFs to adjacent chambers. Cortical neurons were cultured in each chamber. Using this system, the transmission of α-Syn PFFs was monitored via pS129 levels (**Fig 8C**). Administration of α-Syn PFFs to C1 led to increased pS129 levels in C1 and C2 (**Fig 8D**). To assess the propagation of α-Syn PFFs along dendrites and axons as well as transmission of misfolded α-Syn, the levels of pS129 were monitored in C2. When C1 contains 24-OHC treatment, pS129 was increased in C2. When C1 was treated with LAG3 antibody or toyocamycin to inhibit LAG3 or XBP1, pS129 was significantly decreased in C2 **(Fig 8D)**. Taken together, these results indicate that 24-OHC enhances the propagation and transmission of pathologic α-Syn via LAG3 and XBP1 (**Fig 9**).

### Blocking XBP1 ameliorates 24-OHC-induced mitochondrial damage

To confirm the role of XBP1 in the mitochondrial damage induced by α-Syn, SH-SY5Y cells were treated with α-Syn PFFs, 24-OHC and toyocamycin, and TMRE staining was used to detect the mitochondrial membrane potential. The results showed that toyocamycin partially ameliorated the loss of the mitochondrial membrane potential induced by 24-OHC (**S9A Fig**). Moreover, immunoblotting revealed that inhibition of XBP1 decreased the loss of Tom20 and COX-IV (**S9B Fig**). These results suggest that inhibition of XBP1 can alleviate 24-OHC-induced mitochondrial damage, suggesting that the XBP1-LAG3 axis is involved in mitochondrial damage.

## Discussion

Our results indicate that CYP46A1 promotes α-Syn pathology and nigrostriatal dopaminergic degeneration, whereas depletion of CYP46A1 attenuates damage in a mouse model of synucleinopathy. The brain-specific oxysterol 24-OHC, catalyzed by CYP46A1, facilitates the aggregation, propagation, and neurotoxicity of α-Syn. 24-OHC also exacerbates the mitochondrial dysfunction triggered by α-Syn fibrils. Intracerebroventricular injection of 24-OHC exacerbates α-Syn pathology and dopaminergic neuronal degeneration. Moreover, 24-OHC and CYP46A1 activate the XBP1-LAG3 axis and promote neuron-to-neuron transmission of pathological α-Syn. Considering that the levels of 24-OHC are increased in the CSF and plasma of PD patients and that CYP46A1 is overexpressed, aberrant activation of the CYP46A1-24-OHC axis may play a vital role in the pathogenesis of PD.

Clinical evidence indicates that the levels of 24-OHC in the CSF and plasma of PD patients are greater than those in healthy controls and are correlated with the duration of disease [20]. Some studies reported that plasma 24-OHC levels were not altered or even decreased in PD patients [28,29]. There are several possible reasons for these contradictory results. First, dopamine supplementation, which is commonly used in PD patients, has been shown to interfere with lipid levels in patients [30]. Extremely low levels of 24-OHC have been reported in the plasma of PD patients treated with L-DOPA [20,31]. The PD patients in the study of Bjorkhem and colleagues [20] were newly diagnosed and did not receive anti-PD drugs. The studies by Lee and colleagues [29] and Huang and colleagues [28] did not mention whether the patients received medication. In addition, different methodologies for detecting 24-OHC may affect the results. Different internal standards for LC–MS analysis have been used in different studies. The internal standard was d4-24-hydroxycholesterol in our study and in the study by Bjorkhem and colleagues, 24-hydroxycholesterol-25,26,26,26,27,27,27-d7 in the study by Lee and colleagues, and 24 (R/S)-OH-cholesterol-d6 in the study by Huang and colleagues. Another potentially important difference between these studies is the use of different solvents to elute the cholesterol oxidation products from the SPE columns. Moreover, all the existing studies included small numbers of patients. It would be interesting to include a large cohort of drug-naïve PD patients to determine the concentration of 24-OHC in the plasma or even CSF.

A comprehensive lipidomic analysis of several brain regions in patients with PD revealed that the cortex had increased 24-OHC levels and increased CYP46A1 levels [32]. Accordingly, we also found that the levels of CYP46A1 and 24-OHC were increased in the brains of PD patients. Moreover, deletion of CYP46A1 was beneficial in our model. Notably, the levels of 24-OHC were dramatically decreased but still detectable in the brains of CYP46A1^−/−^ mice. This finding is consistent with a previous report in which 24-OHC was detected in the plasma and brain of CYP46A1^−/−^ mice [33]. These results suggest that 24-OHC is primarily catalyzed by CYP46A1, but this is not the only way to produce 24-OHC. Notably, the levels of cholesterol were slightly elevated in the CYP46A1^−/−^ mice. It is possible that altered levels of cholesterol may also contribute to the beneficial effect of CYP46A1 deletion. CYP46A1 inhibition alleviated inflammation, oxidative stress, and N-methyl-D-aspartate (NMDA) signaling [34]. Interestingly, CYP46A1 has been associated with other neurodegenerative diseases, such as Alzheimer’s disease (AD) and Huntington’s disease (HD) [35–37]. Adeno-associated virus-mediated overexpression of CYP46A1 has been shown to exert protective effects in a mouse model of AD [38]. AD is characterized by synaptic impairment [39]. 24-OHC is a very potent positive allosteric modulator of the N-methyl-D-aspartate receptor [17,40]. In hippocampal slices, higher 24-OHC levels increase the ability of subthreshold stimuli to induce long-term potentiation, suggesting that 24-OHC enhances synaptic function [17,40]. Moreover, aged females are more susceptible to AD than are aged males. In aged female mice, overexpression of CYP46A1 significantly enhances estrogen signaling in the hippocampus, leading to improved cognitive function. Conversely, age-matched male mice with CYP46A1 overexpression exhibit anxiety-like behavior, impaired memory, and increased levels of 5α-dihydrotestosterone in the hippocampus [41]. While sex is a significant risk factor for AD, it is not a risk factor for PD. The regulatory role of CYP46A1/24-OHC in the estrogen signaling pathway also explains the fundamentally different effects of the CYP46A1/24-OHC pathway in AD compared with PD. A previous study reported that AAV-mediated delivery of shCYP46A1 into the striatum of wild-type mice reproduced the HD phenotype, whereas AAV-mediated delivery of CYP46A1 into the striatum decreased HD-like phenotypes in a mouse model [35]. The protective effect of CYP46A1 may be attributed to activated autophagy and decreased huntingtin build-up [42]. In PD, the main pathological manifestations are the loss of dopaminergic neurons and α-syn aggregation [43]. Elevated 24-OHC levels have been found to induce neuronal death [44–46]. These results suggest that CYP46A1 may exert distinct effects in different disease conditions. Furthermore, we used CYP46A1 knockout mice, which may trigger compensatory effects that are protective.

It has been reported that 24-OHC induces neuronal cell death by ACAT1-catalyzed esterification of 24-OHC with long-chain unsaturated fatty acids followed by the formation of atypical LD-like structures at the ER membrane [46]. 24-OHC has also been shown to have pro-oxidant activities in PD [44,47]. We first used a mouse model with continuous injection of 24-OHC into the lateral ventricle and α-Syn PFFs into the striatum. We found that intracerebroventricular injection of 24-OHC aggravated LB-like α-Syn pathology, the loss of TH-positive neurons, and behavioral impairments.

Prion-like transmission and propagation of α-Syn aggregates play crucial roles in the development and progression of PD. However, the specific triggers that initiate α-Syn aggregation in the brain remain largely unknown. Our findings suggest that 24-OHC could be a potential modulator of α-Syn assembly. Recent studies have revealed highly heterogeneous properties of these assembled proteins, manifested in various ways [25]: (1) morphological polymorphisms; (2) diverse aggregation kinetics and preferences for propagation and distribution; (3) varying levels of cytotoxicity; and (4) differences in resistance to proteolytic inactivation. We observed that 24-OHC promotes α-Syn assembly, resulting in the formation of morphologically distinct fibrils that are resistant to proteolysis. The considerable heterogeneity of α-Syn fibrils may contribute to the diversity of symptoms observed in PD patients. Notably, α-Syn is constitutively N-terminally acetylated. The N-acetylation of α-Syn affects its aggregation and cytotoxicity [48,49]. We used recombinant α-Syn, which lacks native N-acetylation, in this study. Thus, caution should be used in interpreting the data obtained with recombinant α-Syn.

Converging evidence supports the cell-to-cell transmission of α-Syn pathology [50,51]. LAG3 has been suggested to be a receptor that mediates the spread of α-Syn seeds [14]. In addition, the transcription factor XBP1 regulates the expression of LAG3, although the underlying molecular mechanisms remain unknown [27,52]. We found that the levels of LAG3 increased in the presence of 24-OHC, which was abolished by the XBP1 inhibitor toyocamycin, demonstrating that 24-OHC promotes α-Syn propagation by activating the XBP1–LAG3 axis. We found that LAG3 colocalized with α-syn aggregates in the mouse brain, suggesting that it is sequestered by these aggregates.

Mitochondrial dysfunction plays a key role in neurodegeneration in PD. Loss of functional mitochondrial complex I in the dopaminergic neurons of the SN is a hallmark of PD [53–55]. Similar to what has been observed in the brains of PD patients [56–59], we found that pathological α-Syn colocalized with mitochondria, which was enhanced by 24-OHC. It has been suggested that the binding of α-Syn to lipid-rich domains determines its subcellular localization [60,61]. 24-OHC together with α-Syn PFFs alters mitochondrial function and dynamics. Mitochondrial dysfunction includes complex I deficiency, increased oxidative stress, and lipid abnormalities, ultimately inducing neuronal degeneration [62]. Mutations in genes encoding mitochondrion-associated proteins, including PINK1, parkin, DJ-1, and CHCHD2, have been shown to cause progressive and human-like Parkinsonism, demonstrating that mitochondrial dysfunction is sufficient to induce neuronal injury [63]. Interestingly, we found that more mitochondria were distributed around inclusions than in other regions, indicating that α-Syn inclusions trigger the redistribution of mitochondria. This finding is consistent with previous reports that LBs consist of crowded organelles and lipid membranes [21]. It has been reported that the inhibition of XBP1 attenuates caspase-1 activation and mitochondrial damage, promoting cell survival [64,65]. Consistently, we found that the inhibition of the XBP1/LAG3 axis partially alleviates the mitochondrial damage induced by 24-OHC. These data suggest that aberrant activation of the XBP1/LAG3 axis contributes to the mitochondrial damage induced by 24-OHC.

In summary, our results indicate that the CYP46A1–24-OHC axis contributes to the pathogenesis of PD by promoting the mitochondrial dysfunction induced by α-Syn PFFs. CYP46A1 in the brain is anomalously activated in an age-dependent manner, which explains why aging is the most important risk factor for PD. Moreover, excess 24-OHC and overexpressed CYP46A1 promote the cell-to-cell transmission of α-Syn PFFs by activating the XBP1–LAG3 axis. It will be interesting to determine whether CYP46A1 and 24-OHC can serve as theranostic biomarkers for disease-modifying therapies.

## Materials and methods

### Human plasma

Plasma samples were collected from PD patients and age-matched controls in Renmin Hospital of Wuhan University. Informed consent was obtained from the subjects. The study was approved by the ethical committee of Renmin Hospital, Wuhan University. The approval number is WDRY2024-K104. All participants provided oral informed consent prior to the start of the study. This study was conducted in strict adherence to the ethical principles outlined in the Declaration of Helsinki. PD was diagnosed according to the criteria of the Movement Disorders Society 2015. Disease severity was assessed with Hoehn and Yahr staging (H-Y stage). The plasma was centrifuged, aliquoted, and stored at −80°C.

### Human tissue samples

Postmortem brain samples were dissected from the frozen brains of PD patients and age-matched control cases from the Emory Alzheimer’s Disease Research Center. Age- and sex-matched PD-free samples were used as controls. PD patients were clinically diagnosed and neuropathologically confirmed. The study was approved by the Biospecimen Committee.

### Animals

CYP46A1-knockdown mice on the C57BL/6 background were generated by GemPharmatech (Nanjing, China). Briefly, CRISPR/Cas9 technology was used to modify the mouse *Cyp46a1* gene. The *Cyp46a1* gene has 4 transcripts: cyp46a1-201 (ENSMUST00000021684.5), 202 (ENSMUST00000221708.1), 203 (ENSMUST00000222240.1), and 204 (ENSMUST00000222902.1). According to the gene structure, exons 2–9 of the Cyp46a1-201 transcript were selected as the knockout region. The region contains a 788 bp coding sequence. Knocking out this region results in non-expression of protein. Wild-type (WT), CYP46A1 heterozygous (CYP46A1^**+/−**^), and homozygous (CYP46A1^**−/−**^) littermates were randomly assigned to different treatment groups. The human A53T variant α-syn transgenic line M83 was obtained from the Jackson Laboratory (stock number: 004479). All animal studies were performed in accordance with the Experimental Animal Management Criterion. Experimental procedures were approved by the Institutional Animal Care and Use Committee (IACUC) of Renmin Hospital of Wuhan University with the IACUC issue number of WDRM animal (welfare) 20210609.

### Antibodies and reagents

The following antibodies were used in this study: phospho-α-Syn (pSer129) (1:1,000, Biolegend, 825701), phospho-α-Syn (pSer129) (1: 1,000, Cell Signaling Technology, 23706), α-syn (1:1,000, syn211, Invitrogen, MA5-12272), α-syn (1:1,000, Cell Signaling Technology, 4179), MAP2 (1:1,000, Proteintech, 17490-1-AP), CYP46A1 (1:1,000, Proteintech, 12486-1-AP), XBP1 (1:1,000, Abcam, ab37152), LAG3 (1:1,000, Abcam, ab209236), LAG3 (1:500, Biolegend, C9B7W), and TH (1:2,000, Abcam, ab117112). Alexa Fluor 488-goat anti-mouse (1:500, Invitrogen, A11001), Alexa Fluor 488-goat anti-rabbit (1:500, Invitrogen, A11034), Alexa Fluor 594-goat anti-rabbit (1:500, Invitrogen, A11012), Alexa Fluor 594-goat anti-mouse (1:500, Invitrogen, A11005), anti-mouse IgG-HRP-conjugated detection antibody (1:3,000, Cell Signaling Technology, 7076S), and anti-rabbit IgG-HRP-conjugated detection antibody (1:3000, Cell Signaling Technology, 7074S). The following reagents were used in this study: DAPI (1 μg/ml, BioFroxx, 1155MG010). IHC Detection System Kit (ZSGB-BIO, PV-6001/PV-6002), DAB Kit (ZSGB-BIO, ZLI-9019), MitoTracker (Invitrogen, M7512), Toyocamycin (MedChemExpress, HY-103248), TMRE (Invitrogen, T669), Fluo-4 acetoxymethyl (Fluo-4 AM) ester (Solarbio, IF1500), Lipofectamine 2000 (Invitrogen, 11668019), B-27 (Invitrogen/Gibco, 17504044), L-glutamine (Invitrogen/Gibco, A1286001), penicillin and streptomycin (Invitrogen/Gibco, 10378016), fetal bovine serum (Invitrogen/Gibco, 10099–141), BCA Kit (Invitrogen, A55860), 24-OHC (MedChemExpress, HY-N2370), and α-Syn aggregate ELISA Kit (Biolegend, 449407).

### Purification of recombinant α-Syn

Recombinant mouse α-Syn was generated as previously described [10]. Briefly, His-tagged α-Syn was inserted into the PRK172 plasmid and expressed in *Escherichia coli* strain BL21 (DE3). The cells were cultured in Luria–Bertani broth with 50 μg/ml ampicillin. The cell pellets were collected via centrifugation at 8,000 × rpm and lysed on ice. α-Syn was then purified by His-affinity chromatography and lyophilized. Coomassie blue staining and bicinchoninic acid (BCA) assays were used to determine the purity and concentration of the recombinant α-Syn. α-Syn was stored at −80°C.

### Generation of α-Syn PFFs and 24-OHC PFFs

α-Syn PFFs were generated as previously described [10]. Briefly, the lyophilized protein was dissolved in PBS and centrifuged at 100,000 rpm for 1 h at 4°C with an ultracentrifuge (Beckman Coulter) to obtain high-purity monomeric α-Syn. The supernatant was diluted in PBS to a final volume of 1,000 μl and a final concentration of 2 mg/ml (pH 7.4). α-Syn PFFs were prepared by agitation in a thermomixer for 7 days (1,000 rpm at 37°C) (Eppendorf, Hamburg, Germany). A previous report that the concentration of 24-OHC in brain tissue is 8.6 to 15.1 ng/mg net weight (equal to approximately 30 μm) [24]. 24-OHC PFFs were prepared by adding 30 μm 24-OHC (MCE, HY-N2370) to the α-Syn fibrillization reaction mixture. Aliquots of 5 μl from the incubation mixture were taken at various time points, diluted to 100 μl with 25 μm Thioflavin T (ThT) in PBS, and incubated for 10 min at room temperature. A SpectraMax plate reader (Molecular Devices, Sunnyvale, California, United States of America) was used to detect the fluorescence at 450 nm excitation and 510 nm emission.

### Transmission electron microscopy

To examine the morphological characteristics of different fibrils, 24-OHC PFFs and α-Syn PFFs were adsorbed onto glow-discharged 400 carbon-coated copper grids for 2 min. Following rapid double washing with Tris-HCl (50 mM, pH 7.4), the samples were floated on 2 drops of 0.75% uranyl formate for 30 s. The grids were subsequently air-dried and subjected to imaging via a Hitachi HT7800 transmission electron microscope operating at 80 kV. Image capture and digitization were conducted with an ER-80 charge-coupled device (8 megapixels) employing advanced microscopy techniques.

### Transduction of α-Syn PFFs

Before transduction, the fibrils were sonicated with 60 pulses of 10% power (total of 30, 0.5 s on, 0.5 s off). α-Syn fibrils (140 ng/ml, final concentration) were transduced with Lipofectamine 2000. Briefly, α-Syn fibrils were combined with Opti-MEM (Gibco) to a final volume of 100 μl. Then, 96 μl of Opti-MEM and 4 μl of Lipofectamine 2000 (Invitrogen) were mixed in another tube. The 2 tubes were mixed and incubated for 20 min before being added to the culture medium.

### Cell culture

SH-SY5Y cells and HEK293 cells were obtained from the American Type Culture Collection (ATCC). The cells were tested for mycoplasma contamination before use. HEK293 cells stably expressing α-Syn-GFP (α-Syn-GFP HEK293 cells) were established according to previously described methods [23]. Briefly, HEK293 cells were infected with lentivirus encoding human α-Syn cDNA with a GFP tag at the C-terminus. The cells were subsequently passaged every 3 to 4 days at a 1:4 ratio and exposed to puromycin for selective screening. The cells were cultured in DMEM supplemented with 10% (100 mg/ml) fetal bovine serum, 100 U/ml streptomycin, and 100 U/ml penicillin in a humidified atmosphere containing 5% CO_2_ at 37°C.

### Primary neuronal culture

Primary cortical neurons were prepared at embryonic day 16 and cultured in neurobasal media supplemented with B-27, 0.5 mM L-glutamine, penicillin, and streptomycin on tissue culture plates coated with poly-L-lysine. The neurons were cultured for 5 days in vitro; treated with α-Syn PFFs, 24-OHC (final concentration, 30 μm), control AAVs, or AAV-CYP46A1 (prepared by Brain VTA Co., Ltd., Wuhan, China), and incubated for 9 days. Then, the neurons were fixed and immunostained. Each experiment was performed in duplicate and repeated 3 to 6 times.

### Microfluidic neuronal culture

Microfluidic devices (RD900) were obtained from Xona Microfluidic, LLC (Temecula, California, USA). Approximately 100,000 neurons were plated in each chamber. After being cultured for 5 days in vitro, 0.5 μg of α-syn PFFs was added to chamber 1 (C1), which was supplemented with 24-OHC (final concentration, 30 μm), the LAG3 antibody (50 μg/ml), or toyocamycin (final concentration, 40 nM). To control the direction of flow, a 50 μl difference in media volume was maintained between C1 and chamber 2 (C2). After treatment with α-syn PFFs for 9 days, the neurons were fixed with 4% paraformaldehyde (PFA) in PBS and then processed for immunofluorescence.

### Quantification of the fluorescence intensity of the α-Syn inclusions

After treatment, the α-Syn-HEK293 cells were fixed with 4% PFA (40 mg/ml) for 15 min, after which the soluble protein was permeabilized with 1% TX-100 for 15 min at room temperature. After washing, the cells were stained with 1 μg/ml DAPI for 2 min and imaged via an Olympus inverted fluorescence microscope (Olympus TH4-200, Japan). The fluorescence intensities of the α-Syn inclusions were quantified via ImageJ software (version 2.1.0/1.53c).

### Immunofluorescence

For immunofluorescence, cells and neurons were fixed with 4% PFA for 15 min, followed by permeabilization and blocking with 0.1% Triton X-100 (TX-100)/3% BSA at room temperature for 30 min. Samples were incubated with primary antibodies overnight at 4°C. After being washed, the cells were stained with Alexa Fluor 488/594-conjugated anti-mouse/rabbit secondary antibodies for 2 h at room temperature, followed by incubation with 1 μg/ml DAPI for 2 min. The cells were subsequently imaged via an Olympus inverted fluorescence microscope (Olympus TH4-200, Japan). ImageJ software (version 2.1.0/1.53c) was used for quantification of the signals.

### Immunoblotting

The samples were prepared in loading buffer containing 4% SDS and 5% β-mercaptoethanol, boiled for 10 min at 100°C and then separated on 10% SDS–PAGE gels. The proteins were subsequently transferred to nitrocellulose membranes via a semidry system (Bio-Rad, Switzerland). The membranes were blocked with blocking buffer (5% (w/v) nonfat milk (50 mg/ml) in 1× TBS containing 0.05% (v/v) Tween-20 (TBST)) for 1 h at room temperature and then incubated overnight at 4°C with primary antibody diluted in blocking buffer. After being washed 3 times with TBST, the membranes were incubated with appropriate HRP-conjugated secondary antibodies in blocking buffer for 1 h at room temperature. After 3 washes with TBST, the blots were developed via Western light-enhanced chemiluminescence (ECL).

### Immunohistochemistry

The mice were perfused with PBS and 4% PFA successively. The brain was postfixed in 4% PFA overnight at room temperature. The brain samples were dehydrated through a graded series of ethanol and then embedded in paraffin wax. Five micron-thick brain sections were mounted on glass slides, deparaffinized and then rehydrated via a graded series of ethanol. An IHC detection system kit was used for immunohistochemistry according to the manufacturer’s instructions. Briefly, the brain sections were deparaffinized, rehydrated, and then incubated with 3% (v/v) H_2_O_2_ for 25 min to quench endogenous peroxidase activity. Then, the slides were blocked by incubation in 5% (v/v) normal horse serum for 60 min at room temperature and incubated with primary antibodies overnight at 4°C. The signals were developed via the 3,3′-diaminobenzidine (DAB) peroxidase substrate. The brain sections were imaged via an Olympus inverted fluorescence microscope (Olympus TH4-200, Japan) with 20×, 40×, or 63× objectives.

### LAG3 antibody blocking experiments and XBP1 inhibition

Before α-Syn PFF treatment, anti-LAG3 antibodies (C9B7W, Biolegend) were added to the cell cultures (50 μg/ml), which were subsequently incubated for 30 min [14]. Mouse IgG was used as a negative control. The XBP1 inhibitor toyocamycin was dissolved in DMSO. Toyocamycin (final concentration, 40 nM) was added to the cell cultures, which were subsequently incubated for 48 h.

### α-Syn inclusions colocalized with mitochondria

MitoTracker (final concentration of 500 nM, 37°C for 30 min) was used to stain the mitochondria. Live imaging was performed under a confocal microscope with an excitation wavelength of 579 nm and an emission wavelength of 599 nm.

### Detection of mitochondrial morphology and fusion activity

The morphology of the mitochondria was determined as reported previously [66]. SH-SY5Y cells were transfected with Mitodendra2 plasmids and then exposed to α-Syn PFFs in the presence or absence of 30 μm 24-OHC for 48 h. The cells were imaged via a Leica TCS SP8 confocal microscope. To determine the fusion activity of the mitochondria, the cells were live-imaged via a Leica TCS SP8 confocal microscope. The region of interest within a single cell was fully photon-shifted by exposing the region of interest to 405 nm fluorescence (10% power) for 2 s and observing for another 10 s. The colocalization of the shifted mitochondria (red) with the original mitochondria (green) revealed the fused mitochondria. Images were analyzed via ImageJ. Fusion percentage (%) = fused area (yellow)/shifted area (red).

### Measurement of the mitochondrial membrane potential

The mitochondrial membrane potential was detected via tetramethylrhodamine ethyl ester (TMRE). After treatment with α-Syn PFFs in the presence or absence of 30 μm 24-OHC for 48 h, the SH-SY5Y cells were incubated with 150 nM TMRE at 37°C for 30 min. Then, the cells were washed 3 times with PBS. Live imaging was performed under a confocal microscope with an excitation wavelength of 488 nm and an emission wavelength of 575 nm. Images were analyzed via ImageJ software (version 2.1.0/1.53c).

### Calcium imaging

The fluorescent calcium indicator Fluo-4 acetoxymethyl (Fluo-4 AM) ester (Solarbio, IF1500) was used to monitor the levels of intracellular calcium. After treatment, the SH-SY5Y cells were incubated with Fluo-4 AM for 30 min at a final concentration of 1 μm. After 3 washes with Hank’s balanced salt solution (HBSS) (with 2 mM calcium chloride), the cells were imaged under a confocal microscope. Live imaging was performed with an excitation wavelength of 492 nm and an emission wavelength of 514 nm. Images were acquired at 30-s intervals and analyzed via ImageJ software (version 2.1.0/1.53c).

### Stereotaxic injection

Three-month-old mice were anesthetized. PBS containing recombinant α-syn PFFs or 24-OHC PFFs (5 μg/2 μl) was stereotactically delivered into the right striatum. The following reference coordinates for the dorsal neostriatum were used: + 2.0 mm medial–lateral (ML), + 2.6 mm dorsoventral (DV), and + 0.2 mm antero–posterior (AP) from bregma. Injections were performed via a 10 μl syringe (Hamilton, Reno, Nevada, USA) at a rate of 0.3 μl per minute. The needle was left in place for 8 min before being slowly removed. After surgery, the animals were monitored and postsurgical care was provided. The mice were euthanized for biochemical and histological analysis at 30 dpi, 90 dpi, and 180 dpi.

### Intracerebroventricular injection of 24-OHC

Three-month-old mice were unilaterally injected with 5 μg of α-syn PFFs and allowed to rest for 1 month. Then, 24-OHC (100 μg/kg body weight) or vehicle was injected into the right lateral ventricle 3 times a week for 8 weeks [67]. This dose was chosen on the basis of a previous report [68]. 24-OHC was dissolved in 30% (2-hydroxypropyl)-β-cyclodextrin. The same solution was used as the vehicle control. 24-OHC or vehicle was injected into the right lateral ventricle at the following stereotaxic coordinates: 1.0 mm lateral, 2.3 mm ventral, and 0.46 mm from the bregma.

### Quantification of TH-positive neurons

The number of TH-positive neurons in the SNpc was determined via stereological measurements as previously described [9]. The entire substantia nigra of each mouse was continuously sliced into 4 μm thick paraffin sections (100 to 150 sections for each mouse). Every fifth section from the caudal to the rostral boundaries of the SNpc was measured (approximately 20 to 25 sections for each mouse). Each group contained 6 mice. The number of TH-positive neurons was counted by an Olympus DP80 microscope and its matched software (CellSens Ver1.7.1/1.8/1.9 controlling DP80). An investigator who was blinded to the treatment groups performed the cell counting.

### Optical densitometry analysis

Sections through the whole striatum at 6 coronal levels of equal distance relative to the bregma were stained with TH. Optical densitometry was performed with ImageJ software (version 2.1.0/1.53c). The staining signal was calibrated by subtracting the baseline of the cortex.

### Proteinase K (PK) digestion assays

For PK digestion, the brain sections were incubated with PK at a final concentration of 10 μg/ml at 37°C for 45 min. Digestion was terminated by the addition of a protease inhibitor cocktail. The sections were then analyzed via immunohistochemistry.

### Behavioral analysis

To evaluate behavioral deficits, the mice were subjected to the pole test, grip test, balance beam test, and rotarod test 1 week prior to sacrifice. The experimenter was blinded to the treatment groups or genotypes for all behavioral tests. All tests were conducted between 10:00 and 16:00. In the pole test, a metal rod 45-cm long and 1 cm in diameter wrapped with bandage gauze was used as the pole. The mice were placed on the top of the pole facing head-up. The time taken to turn and the total time taken to reach the base of the pole were recorded. The end of the test was defined by the placement of all 4 paws on the base. Before the test, the mice were trained for 2 consecutive days, and each training session consisted of three test trials. The maximum cutoff time to stop the test was 60 s. The turn and total time (in seconds) were recorded. In the grip test, the metal grip was lightly shaken so that the mouse could grab the grip and then turn upside down. The latency of the mice to fall off the grid was recorded. Each training session consisted of 3 test trials. The maximum cutoff time to stop the test was 5 min. Each mouse was examined 3 times. In the rotarod test, the mice were placed on a rotating cylinder with uniform acceleration, rising from 4 rpm to a maximum of 40 rpm in 300 s. The latency of the mice to fall off the rotating cylinder was measured. The maximum cutoff time to stop the test and recording was 300 s. Each mouse was examined 3 times. The average time of the 3 trials was recorded. A narrow beam (2 × 100 cm) was used in the balance beam test. The mice walked along the beam. The time to cross the beam or drop off the beam was measured. Each mouse was examined 3 times. The average time of the 3 trials was recorded.

### Detection of DA and DOPAC in the striatum

HPLC was used to measure the concentrations of biogenic amines. Briefly, the ipsilateral striatum was lysed and sonicated in ice-cold 0.01 mM perchloric acid containing 0.01% EDTA. Then, the lysates were centrifuged at 15,000 × rpm for 15 min. The supernatants were filtered through a 0.2 μm filter and analyzed by HPLC. Thirty microliters of the supernatant was analyzed on an HPLC column (4.6 mm × 150 mm, C-18 reversed-phase column, Acclaim TM Polar Advantage II, Thermo Scientific) by a dual channel coulochem III electrochemical detector (Model 5300, ESA, Inc. Chelmsford, Massachusetts, USA). A BCA protein assay kit was used to measure the protein concentration of the tissue homogenates. The data were normalized to protein concentrations and expressed in ng/mg protein.

### Detection of 24-OHC and cholesterol levels in brain tissue and plasma

The concentrations of 24-OHC were determined by Sensichip Biotech (Sensichip Biotech Co., Ltd., Shanghai, China). The brain tissues were washed 3 times with PBS to remove contaminating blood. The brain and plasma samples were stored at −80°C before being analyzed. Briefly, brain tissues were mixed with 1.5 ml of chloroform/methanol (2/1, v/v) solution, swirled for 1 min, mixed with 0.5 ml of water, and ultrasonicated at 4°C for 30 min. The brain lysates were centrifuged at 12,000 × rpm and 4°C for 10 min. D4-24-hydroxycholesterol was used as an internal standard. The supernatants were used to detect 24-OHC and cholesterol levels via liquid chromatography–mass spectrometry (LC–MS) analysis. The quantification of 24-OHC and cholesterol was performed via the internal standard ratio method.

### Lipidomic analysis

Lipidomic analysis was performed by Shanghai Minsheng Biotechnology, Shanghai, China as previously described [69]. The samples were lipid-extracted with a 2:1 mixture of chloroform/methanol (v/v). The samples were centrifuged, and the organic phase was transferred to an EP tube for rotational evaporation. The dried phase was added to a 1:1 methanol/isopropyl alcohol (v/v) mixture, followed by centrifugation, and the supernatant was collected for detection. LPC (12:0) was used as the internal standard. An Ultimate 3000 LC Phenomenex Kinetex C18 column was subsequently used to separate the samples. The mobile phase consisted of ultrapure water-acetonitrile (40:60, v/v) with ammonium formate and isopropanol-acetonitrile (90:10, v/v) with ammonium formate. For data processing, the raw LC–MS data for all the samples were initially processed via Thermo Lipid Search qualitative analysis software (Thermo Scientific, Waltham, Massachusetts, USA).

### GST pull-down assay

To determine whether CYP46A1 directly interacts with α-Syn, HEK293 cells were co-transfected with GST-tagged α-Syn together with GFP-tagged tau or His-tagged CYP46A1 for 48 h. Then, the cells were collected and lysed with NP-40 for 30 min on ice. The lysates were incubated with glutathione agarose overnight at 4°C. The beads were subsequently washed 4 times with NP-40, boiled in SDS loading buffer, and analyzed by immunoblotting.

### Statistical analysis

All statistical analyses were performed using GraphPad Prism (GraphPad Software Inc., San Diego, California, United States, version 8.0) with a significance threshold of *P* = 0.05. Results were expressed as means ± SEM. One-way ANOVA was performed to confirm the significant main effects and differences among groups, followed by Tukey’s multiple comparisons for post hoc tests. *P* values of <0.05 were considered to be statistically significant.

## Supporting information

S1 Fig CYP46A1 levels are increased in wild-type mice at different ages. Immunoblots showing CYP46A1 levels in the striatal lysates of wild-type mice at different ages. GAPDH was used as the loading control (*n* = 6 mice per group). All data are means ± SEM. One-way ANOVA with Tukey’s multiple comparisons test. ****P < 0.0001, and ns, not significant. Underlying data can be found in S1 Data. The uncropped blots are included in S1 Raw Images.
(TIF)

S2 Fig CYP46A1 and 24-OHC levels in CYP46A1 knockout mice. **(A)** Immunoblot analysis of CYP46A1 in the striatum of wild-type (WT), CYP46A1^+/−^, and CYP46A1^−/−^ mice (*n* = 6 independent experiments). **(B)** Plasma 24-OHC levels detected by LC-MS (*n* = 4–9 mice per group). **(C)** 24-OHC levels in the brain detected by LC-MS (*n* = 4–6 mice per group). All data are means ± SEM. One-way ANOVA with Tukey’s multiple comparisons test. *P < 0.05, **P < 0.01, ****P < 0.0001. Underlying data can be found in S1 Data. The uncropped blots are included in S1 Raw Images.
(TIF)

S3 Fig Cholesterol and other lipid levels in CYP46A1 knockout mice. **(A)** Lipidomic analysis of the striatum of wild-type (WT) and CYP46A1^−/−^ mice (*n* = 4 mice per group). (B) Total cholesterol levels in the striatum of WT and CYP46A1^−/−^ mice determined by LC-MS (*n* = 6 mice per group). Underlying data can be found in S1 Data.
(TIF)

S4 Fig pS129 levels in CYP46A1 knockout mice. pS129 signal quantification on the ipsilateral side at 180 dpi (*n* = 6 mice per group). STR: striatum. SN: substantia nigra. DMV: dorsal motor nucleus of the vagus nerve. All data are means ± SEM. One-way ANOVA with Tukey’s multiple comparisons test. ****P < 0.0001, ns: not significant. Underlying data can be found in S1 Data.
(TIF)

S5 Fig CYP46A1 promotes α-Syn aggregation. α-Syn-HEK293 cells were transduced with α-Syn PFFs together with His-tagged CYP46A1 for 48 h. Fluorescence microscopy images showing aggregates (arrow). Scale bar, 20 mm.
(TIF)

S6 Fig CYP46A1 has no direct interaction with α-Syn. **(A)** HEK293 cells were co-transfected with GST-tagged α-Syn together with GFP-tagged tau or His-tagged CYP46A1 for 48 h. GST pull-down assay was subsequently conducted. **(B)** GST pull-down assay showing the interaction between α-Syn and CYP46A1. The uncropped blots are included in S1 Raw Images.
(TIF)

S7 Fig 24-OHC accelerates the seeding activity of α-Syn fibrils in vitro. α-Syn-HEK293 cells transduced with α-Syn PFFs or 24-OHC PFFs for 48 h. **(A)** Insoluble inclusions (green) and pS129 (red) after the soluble α-Syn species were eliminated using 1% Triton X-100 for 30 min. **(B)** Immunoblots of phosphorylated α-Syn (pS129) in α-Syn-HEK293 cells treated with α-Syn PFFs or 24-OHC PFFs. All data are means ± SEM. One-way ANOVA with Tukey’s multiple comparisons test. ***P < 0.001 and ****P < 0.0001. Underlying data can be found in S1 Data. The uncropped blots are included in S1 Raw Images.
(TIF)

S8 Fig Intracerebroventricular injection of 24-OHC aggravates neurodegeneration in vivo. Images of DAPI staining in the striatum of mice injected with 24-OHC or vehicle. Scale bar, 20 μm.
(TIF)

S9 Fig Blocking XBP1 ameliorates 24-OHC-induced mitochondrial damage. **(A, B)** SH-SY5Y cells were treated with an inhibitor of XBP1 (toyocamycin) for 24 h, followed by exposure to α-Syn PFFs and 24-OHC for 24 h. **(A)** Representative images of TMRE staining. *n* = 6 independent experiments. Scale bar, 20 μm. AU, arbitrary unit. **(B)** Immunoblots and quantification of Tom20 and COX-IV. *n* = 6 independent experiments. All data are means ± SEM. One-way ANOVA with Tukey’s multiple comparisons test. *n* = 6 independent experiments. ****P < 0.0001. Underlying data can be found in S1 Data. The uncropped blots are included in S1 Raw Images.
(TIF)

S1 Table Clinical information of PD patients and control subjects in Fig 1. (DOCX)

S1 Data Statistical data. (XLSX)

S1 Raw Images Uncropped immunoblot images. (PDF)

## Abbreviations

AD: Alzheimer’s disease
ATCC: American Type Culture Collection
BBB: blood–brain barrier
BCA: bicinchoninic acid
CSF: cerebral spinal fluid
DMV: dorsal motor nucleus of the vagus
dpi: days postinjection
HBSS: Hank’s balanced salt solution
HD: Huntington’s disease
HPLC: high-performance liquid chromatography
LAG3: lymphocyte-activation gene 3
LB: Lewy body
LC–MS: liquid chromatography–mass spectrometry
LN: Lewy neurite
NMDA: N-methyl-D-aspartate
PD: Parkinson’s disease
PFA: paraformaldehyde
PFF: preformed fibril
SN: substantia nigra
SNpc: substantia nigra pars compacta
TEM: transmission electron microscopy
WT: wild type
XBP1: X-box binding protein 1
