# Supplementary figures and images for: The cholesterol 24-hydroxylase CYP46A1 promotes α-synuclein pathology in Parkinson’s disease
Source: PLoS Biol. 2025 Feb 18;23(2):e3002974. doi: 10.1371/journal.pbio.3002974 (PMC11835240; doi:10.1371/journal.pbio.3002974)

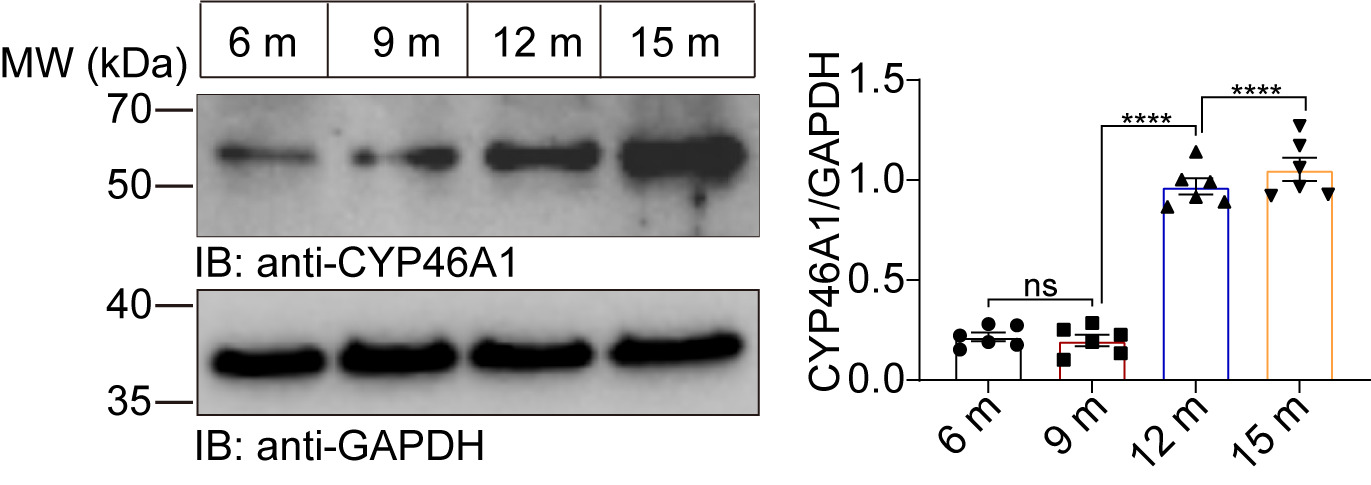

Supplement: S1 Fig — Immunoblots showing CYP46A1 levels in the striatal lysates of wild-type mice at different ages. GAPDH was used as the loading control (n = 6 mice per group). All data are means ± SEM. One-way ANOVA with Tukey’s multiple comparisons test. ****P < 0.0001, and ns, not significant. Underlying data can be found in S1 Data. The uncropped blots are included in S1 Raw Images. (TIF) [file pbio.3002974.s001.tif]

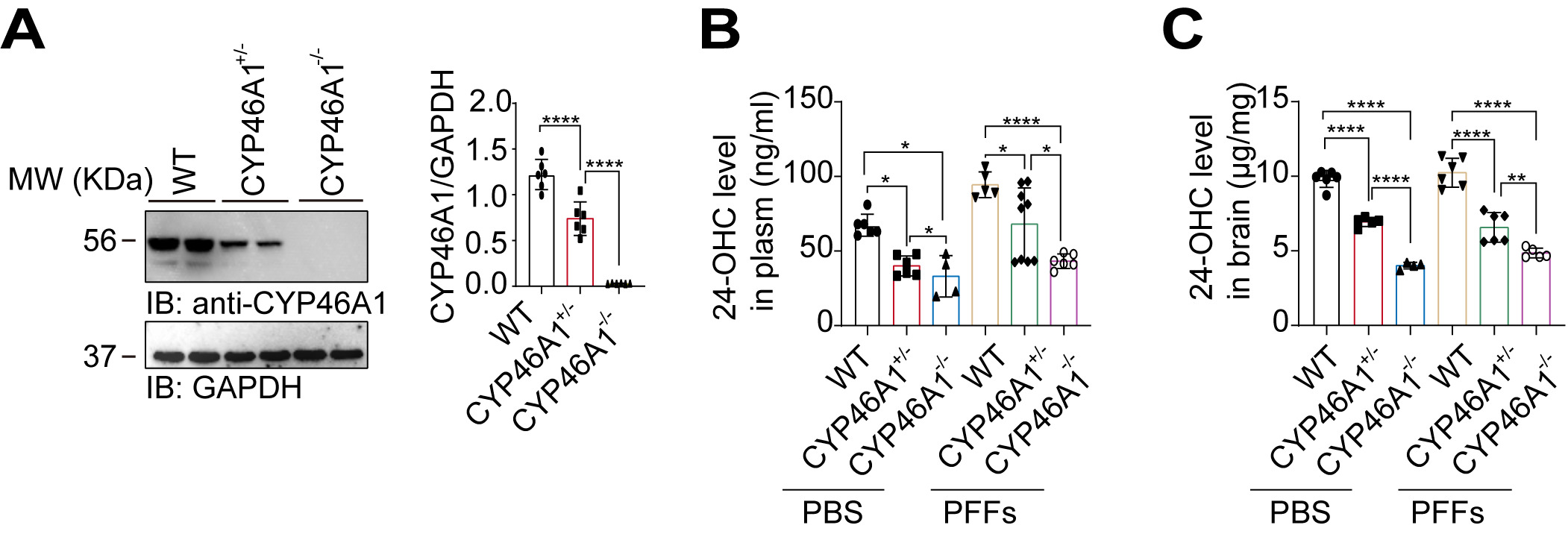

Supplement: S2 Fig — (A) Immunoblot analysis of CYP46A1 in the striatum of wild-type (WT), CYP46A1+/−, and CYP46A1−/− mice (n = 6 independent experiments). (B) Plasma 24-OHC levels detected by LC-MS (n = 4–9 mice per group). (C) 24-OHC levels in the brain detected by LC-MS (n = 4–6 mice per group). All data are means ± SEM. One-way ANOVA with Tukey’s multiple comparisons test. *P < 0.05, **P < 0.01, ****P < 0.0001. Underlying data can be found in S1 Data. The uncropped blots are included in S1 Raw Images. (TIF) [file pbio.3002974.s002.tif]

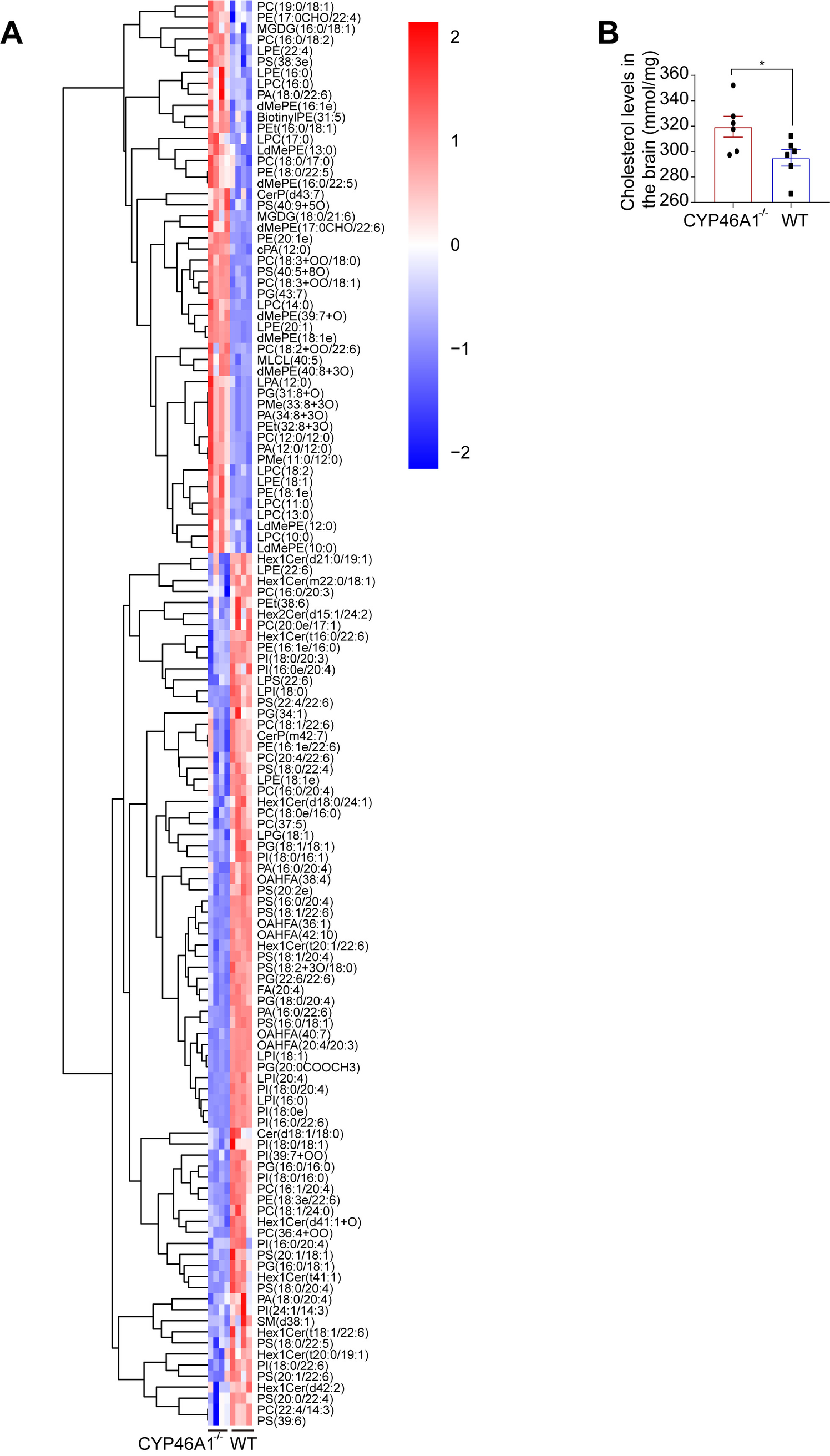

Supplement: S3 Fig — (A) Lipidomic analysis of the striatum of wild-type (WT) and CYP46A1−/− mice (n = 4 mice per group). (B) Total cholesterol levels in the striatum of WT and CYP46A1−/− mice determined by LC-MS (n = 6 mice per group). Underlying data can be found in S1 Data. (TIF) [file pbio.3002974.s003.tif]

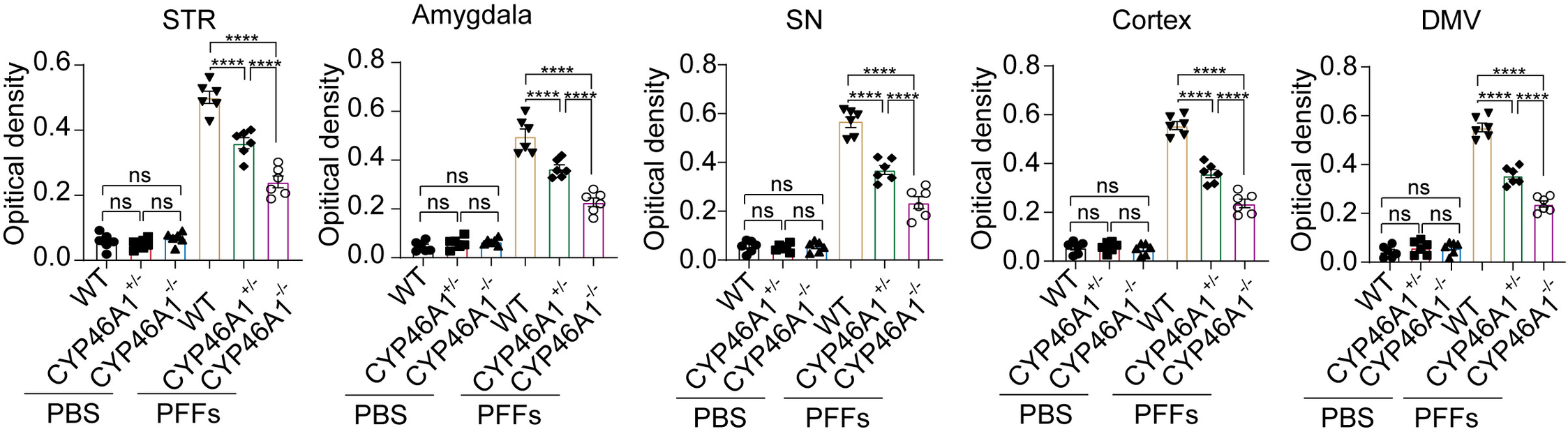

Supplement: S4 Fig — pS129 signal quantification on the ipsilateral side at 180 dpi (n = 6 mice per group). STR: striatum. SN: substantia nigra. DMV: dorsal motor nucleus of the vagus nerve. All data are means ± SEM. One-way ANOVA with Tukey’s multiple comparisons test. ****P < 0.0001, ns: not significant. Underlying data can be found in S1 Data. (TIF) [file pbio.3002974.s004.tif]

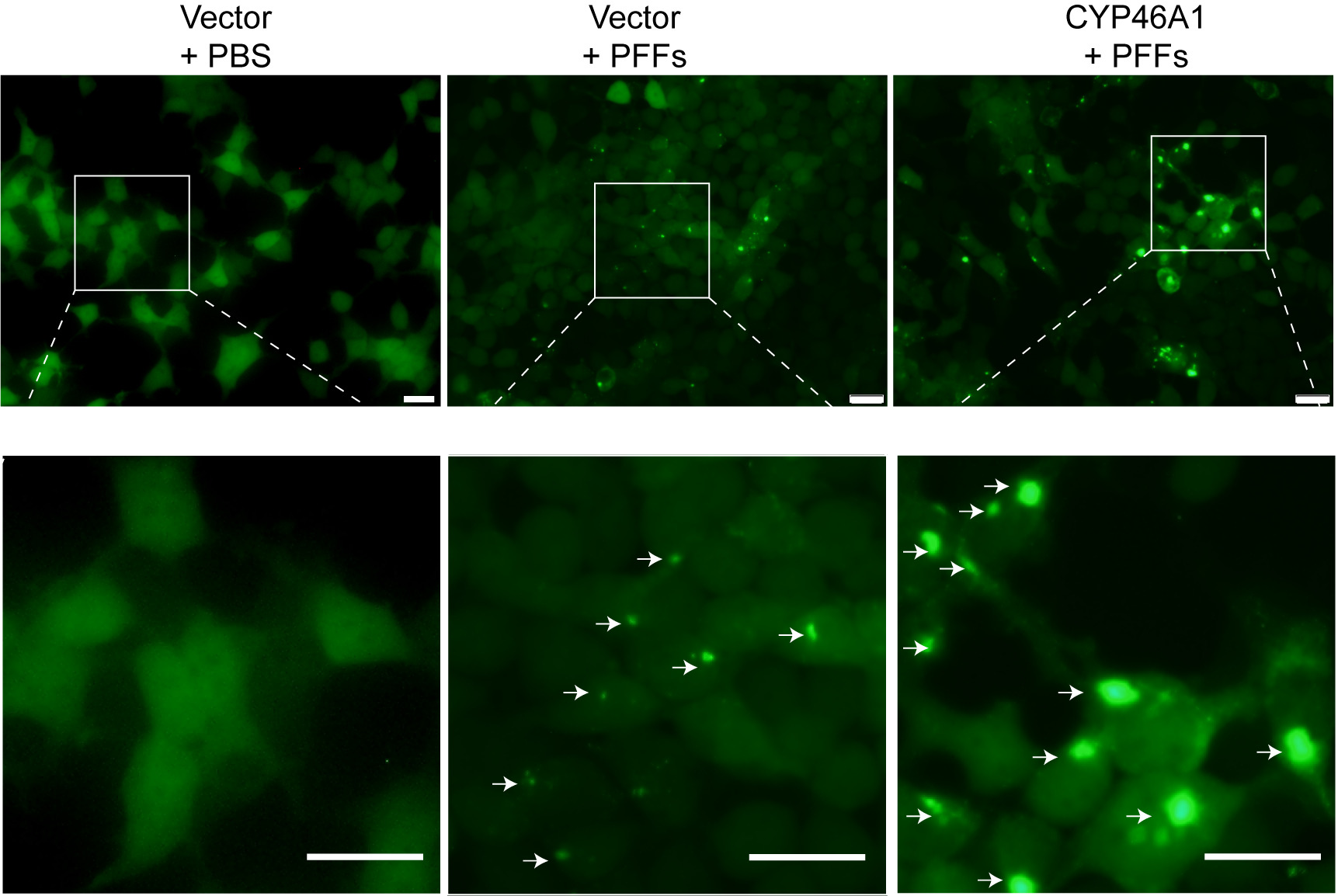

Supplement: S5 Fig — α-Syn-HEK293 cells were transduced with α-Syn PFFs together with His-tagged CYP46A1 for 48 h. Fluorescence microscopy images showing aggregates (arrow). Scale bar, 20 mm. (TIF) [file pbio.3002974.s005.tif]

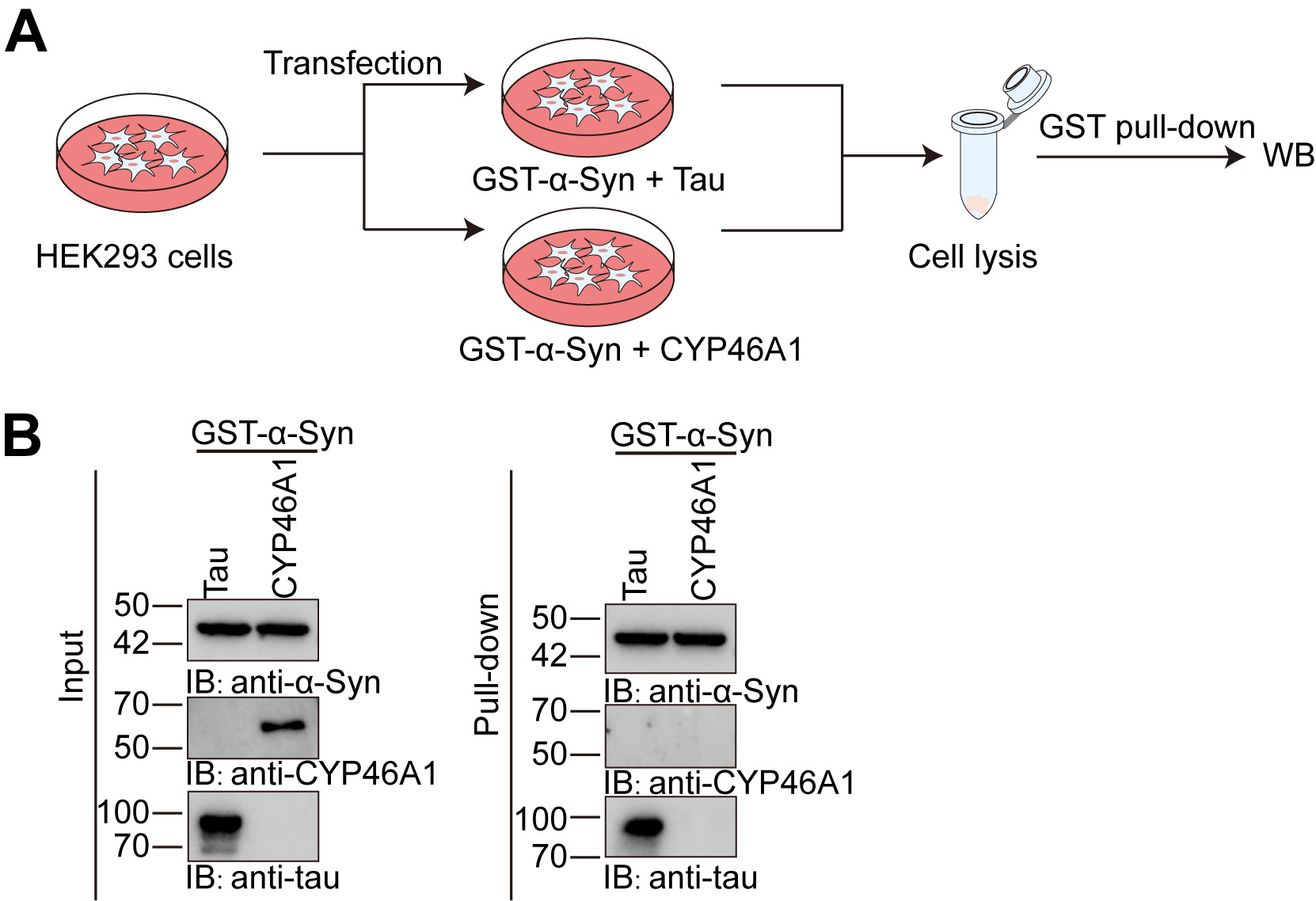

Supplement: S6 Fig — (A) HEK293 cells were co-transfected with GST-tagged α-Syn together with GFP-tagged tau or His-tagged CYP46A1 for 48 h. GST pull-down assay was subsequently conducted. (B) GST pull-down assay showing the interaction between α-Syn and CYP46A1. The uncropped blots are included in S1 Raw Images. (TIF) [file pbio.3002974.s006.tif]

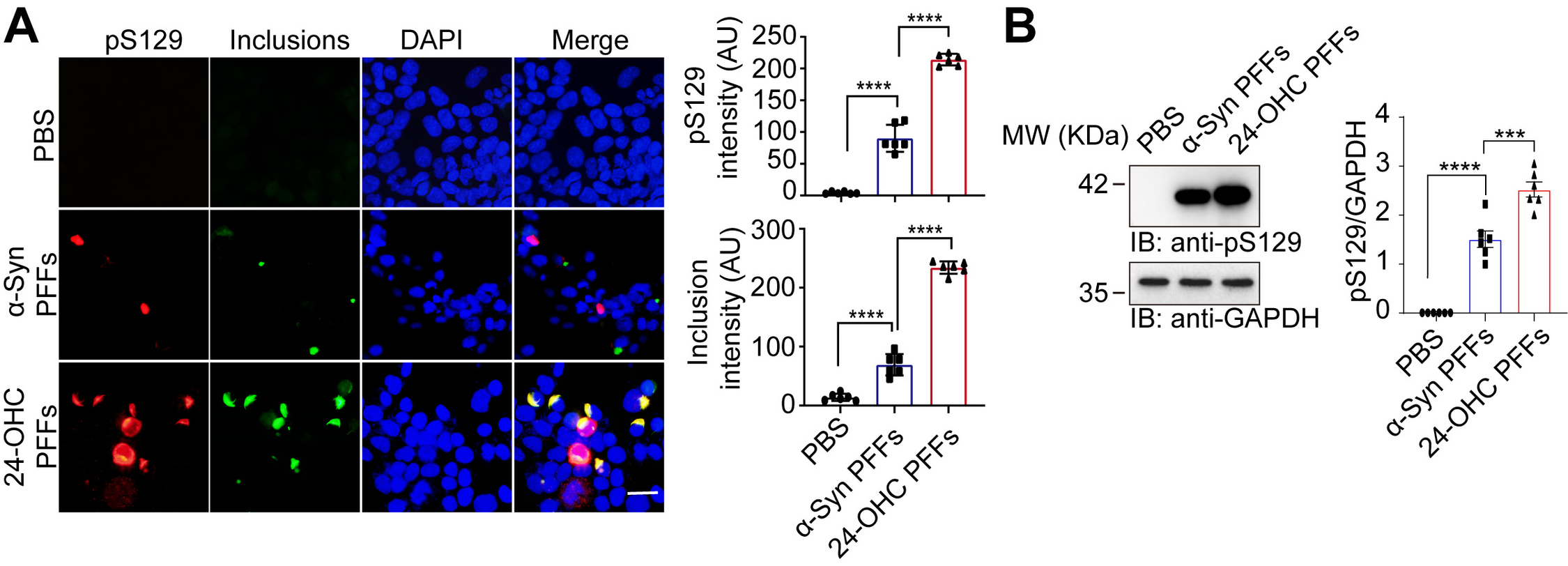

Supplement: S7 Fig — α-Syn-HEK293 cells transduced with α-Syn PFFs or 24-OHC PFFs for 48 h. (A) Insoluble inclusions (green) and pS129 (red) after the soluble α-Syn species were eliminated using 1% Triton X-100 for 30 min. (B) Immunoblots of phosphorylated α-Syn (pS129) in α-Syn-HEK293 cells treated with α-Syn PFFs or 24-OHC PFFs. All data are means ± SEM. One-way ANOVA with Tukey’s multiple comparisons test. ***P < 0.001 and ****P < 0.0001. Underlying data can be found in S1 Data. The uncropped blots are included in S1 Raw Images. (TIF) [file pbio.3002974.s007.tif]

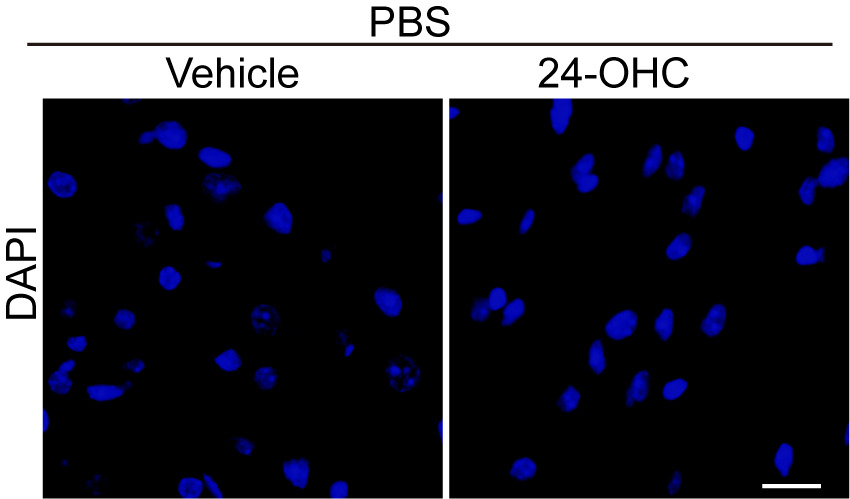

Supplement: S8 Fig — Images of DAPI staining in the striatum of mice injected with 24-OHC or vehicle. Scale bar, 20 μm. (TIF) [file pbio.3002974.s008.tif]

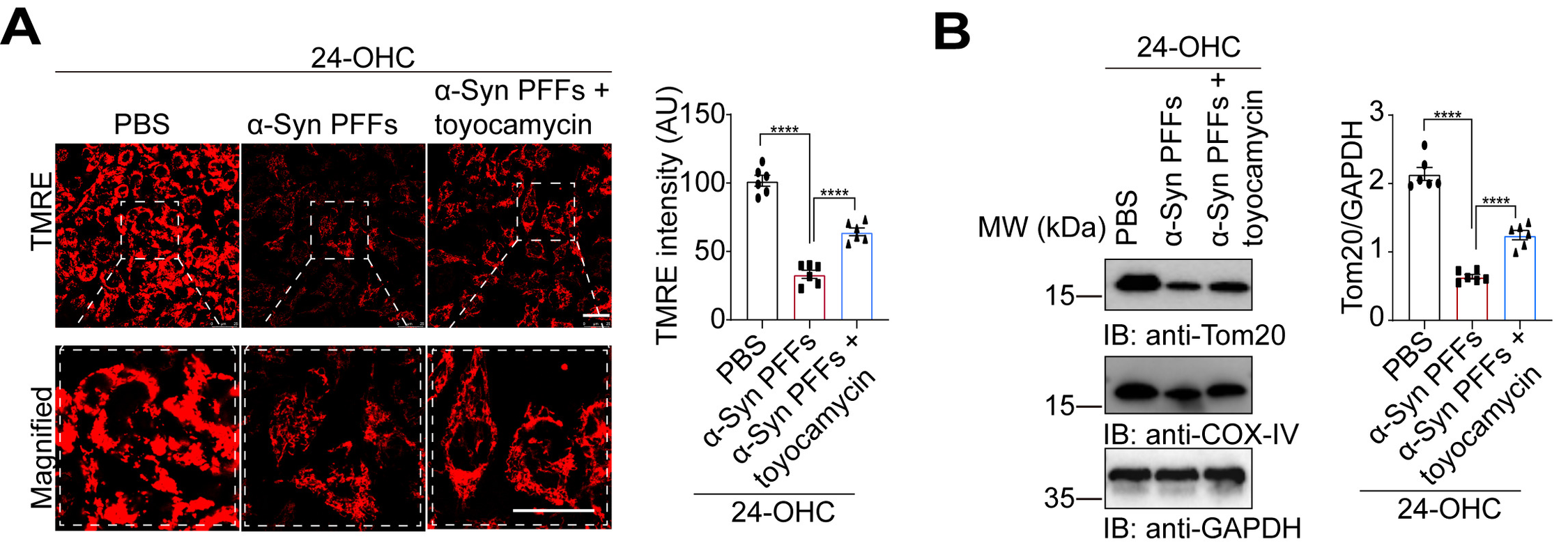

Supplement: S9 Fig — (A, B) SH-SY5Y cells were treated with an inhibitor of XBP1 (toyocamycin) for 24 h, followed by exposure to α-Syn PFFs and 24-OHC for 24 h. (A) Representative images of TMRE staining. n = 6 independent experiments. Scale bar, 20 μm. AU, arbitrary unit. (B) Immunoblots and quantification of Tom20 and COX-IV. n = 6 independent experiments. All data are means ± SEM. One-way ANOVA with Tukey’s multiple comparisons test. n = 6 independent experiments. ****P < 0.0001. Underlying data can be found in S1 Data. The uncropped blots are included in S1 Raw Images. (TIF) [file pbio.3002974.s009.tif]
