# Supplementary material for: The cholesterol 24-hydroxylase CYP46A1 promotes α-synuclein pathology in Parkinson’s disease
Source: PLoS Biol. 2025 Feb 18;23(2):e3002974. doi: 10.1371/journal.pbio.3002974 (PMC11835240; doi:10.1371/journal.pbio.3002974)
Supplement: S1 Table — (DOCX) [file pbio.3002974.s010.docx]

**S1 Table. Clinical information of PD patients and control subjects in Fig 1**

| PD patients  (n = 38) | Sex | Age (year) | Disease duration (year) | Hoehn & Yahr |
| --- | --- | --- | --- | --- |
|  | F | 64 | 5 | 2 |
|  | F | 65 | 8 | 2.5 |
|  | F | 53 | 6 | 2 |
|  | F | 79 | 2 | 2 |
|  | F | 76 | 8 | 3 |
|  | F | 63 | 5 | 2 |
|  | F | 54 | 3 | 2 |
|  | F | 66 | 4 | 2.5 |
|  | F | 78 | 12 | 4 |
|  | F | 74 | 2 | 1 |
|  | F | 83 | 7 | 4 |
|  | F | 66 | 6 | 2 |
|  | F | 69 | 8 | 3 |
|  | F | 70 | 4 | 2 |
|  | F | 56 | 2 | 2 |
|  | F | 77 | 3 | 2 |
|  | F | 65 | 7 | 3 |
|  | F | 76 | 3 | 2 |
|  | F | 67 | 2 | 2 |
|  | F | 52 | 8 | 2 |
|  | F | 57 | 14 | 4 |
|  | M | 66 | 2 | 2.5 |
|  | M | 73 | 6 | 2 |
|  | M | 58 | 2 | 1 |
|  | M | 84 | 3 | 1 |
|  | M | 65 | 3 | 2 |
|  | M | 69 | 3 | 2 |
|  | M | 53 | 1 | 2 |
|  | M | 57 | 2 | 1 |
|  | M | 49 | 5 | 2 |
|  | M | 72 | 2 | 3 |
|  | M | 47 | 1 | 1 |
|  | M | 78 | 2 | 2.5 |
|  | M | 58 | 2 | 2 |
|  | M | 49 | 7 | 2.5 |
|  | M | 80 | 5 | 2 |
|  | M | 65 | 6 | 2 |
|  | M | 68 | 5 | 3 |
| Control  (n = 19) | F | 54 | 0 | 0 |
|  | F | 75 | 0 | 0 |
|  | F | 56 | 0 | 0 |
|  | F | 74 | 0 | 0 |
|  | F | 72 | 0 | 0 |
|  | F | 73 | 0 | 0 |
|  | F | 52 | 0 | 0 |
|  | F | 60 | 0 | 0 |
|  | F | 58 | 0 | 0 |
|  | F | 50 | 0 | 0 |
|  | M | 62 | 0 | 0 |
|  | M | 55 | 0 | 0 |
|  | M | 67 | 0 | 0 |
|  | M | 68 | 0 | 0 |
|  | M | 61 | 0 | 0 |
|  | M | 54 | 0 | 0 |
|  | M | 61 | 0 | 0 |
|  | M | 68 | 0 | 0 |
|  | M | 78 | 0 | 0 |
| **Summarized information** | | | | |
| Group | Mean age ± *SEM* | | Male/female ratio (%) | Mean disease duration ± *SEM* |
| PD | 65.82 ± 10.13 | | 44.7/55.3 | 2.224 ± 0.7596 |
| Control | 63.05 ± 8.644 | | 47.4/52.6 | 0 |

* Disease duration is the time since PD symptom onset.
