# Supplementary material for: The cholesterol 24-hydroxylase CYP46A1 promotes α-synuclein pathology in Parkinson’s disease
Source: PLoS Biol. 2025 Feb 18;23(2):e3002974. doi: 10.1371/journal.pbio.3002974 (PMC11835240; doi:10.1371/journal.pbio.3002974)

Fig. 1-D

6 m | 9 m | 12 m | 15 m

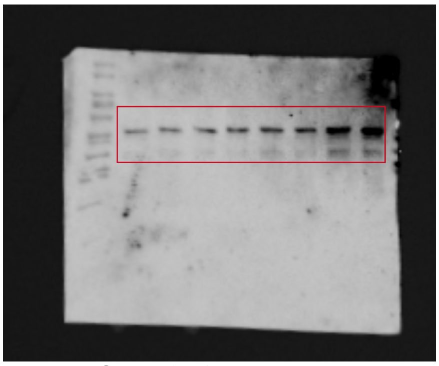

IB: anti-CYP46A1

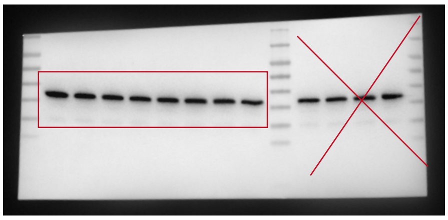

IB: anti-GAPDH

Fig. 2-D

PBS: WT, CYP46A1$^{+/-}$, CYP46A1$^{-/-}$ — α-Syn PFFs: WT, CYP46A1$^{+/-}$, CYP46A1$^{-/-}$

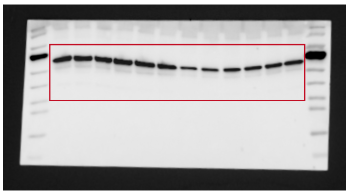

IB: anti-TH

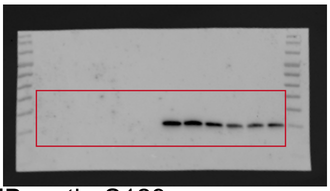

IB: anti-pS129

PBS: WT, CYP46A1$^{+/-}$, CYP46A1$^{-/-}$ — α-Syn PFFs: WT, CYP46A1$^{+/-}$, CYP46A1$^{-/-}$

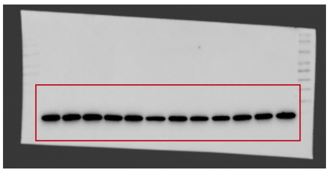

IB: anti-α-Syn

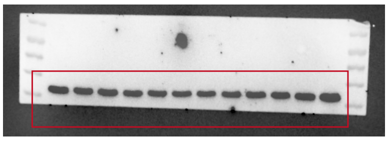

IB: anti-GAPDH

Fig. 4-G

MW (kDa) — PBS | α-Syn PFFs | 24-OHC PFFs

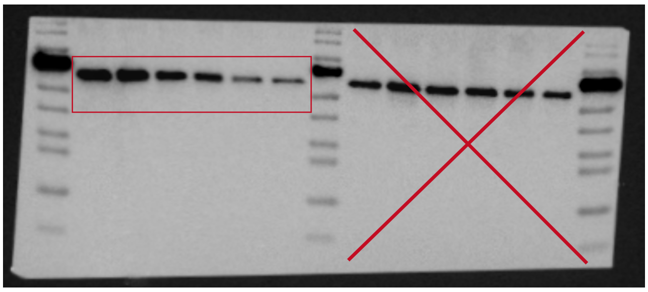

IB: anti-TH

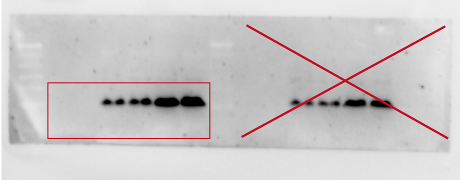

IB: anti-pS129

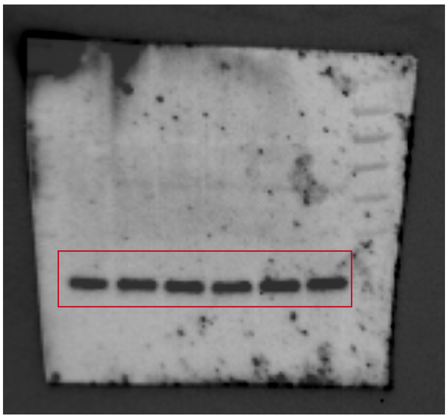

IB: anti-α-Syn

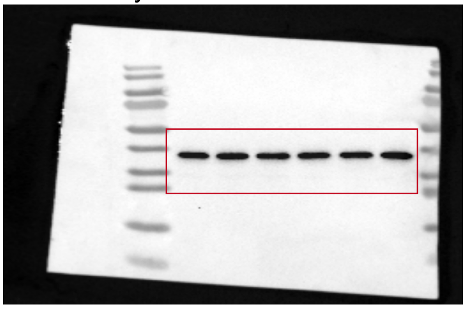

IB: anti-GAPDH

Fig. 3-B

α-Syn PFFs: Vector, Vector, CYP46A1

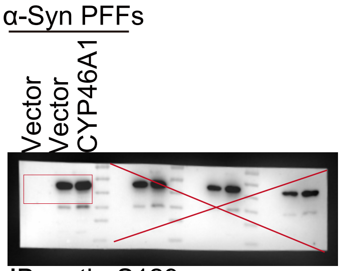

IB: anti-pS129

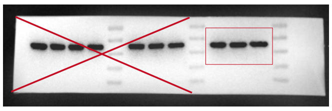

IB: anti-α-Syn

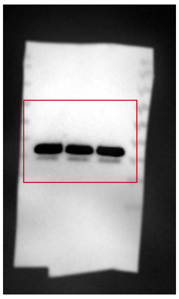

IB: anti-GAPDH

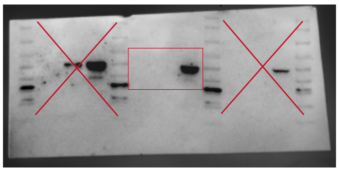

IB: anti-CYP46A1

Fig. 5-B

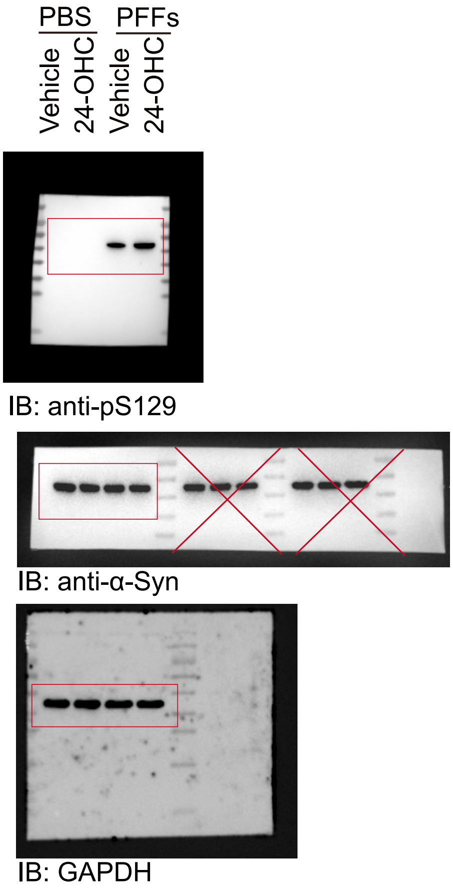

Fig. 6-F

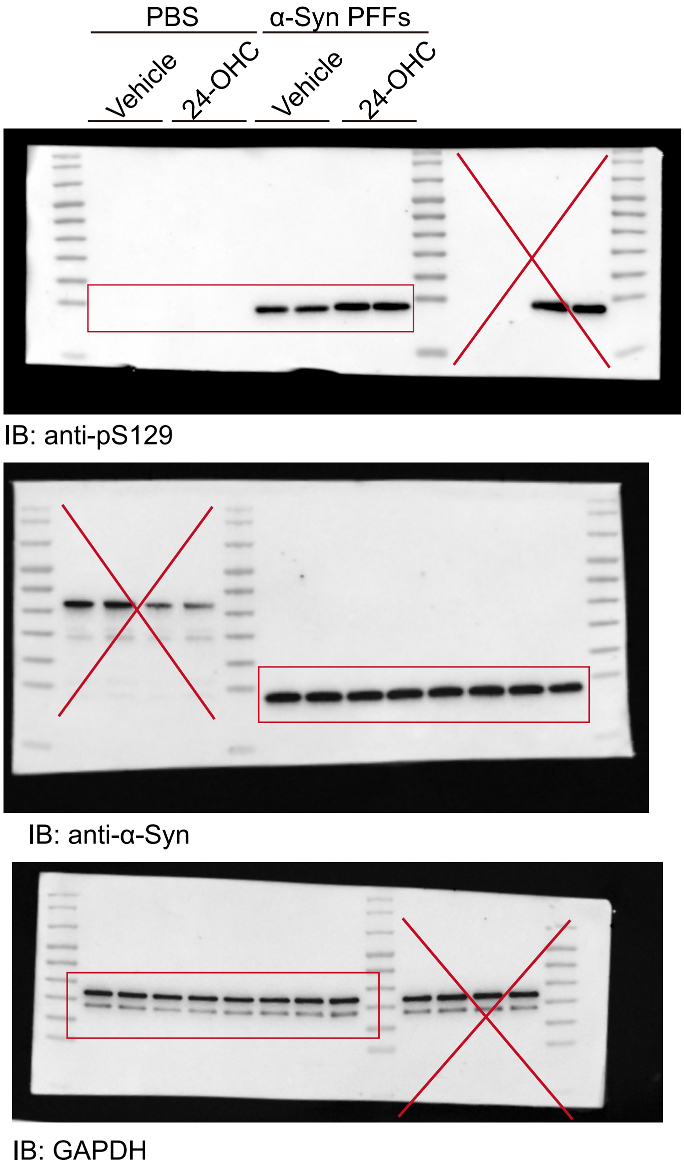

Fig. 7-A

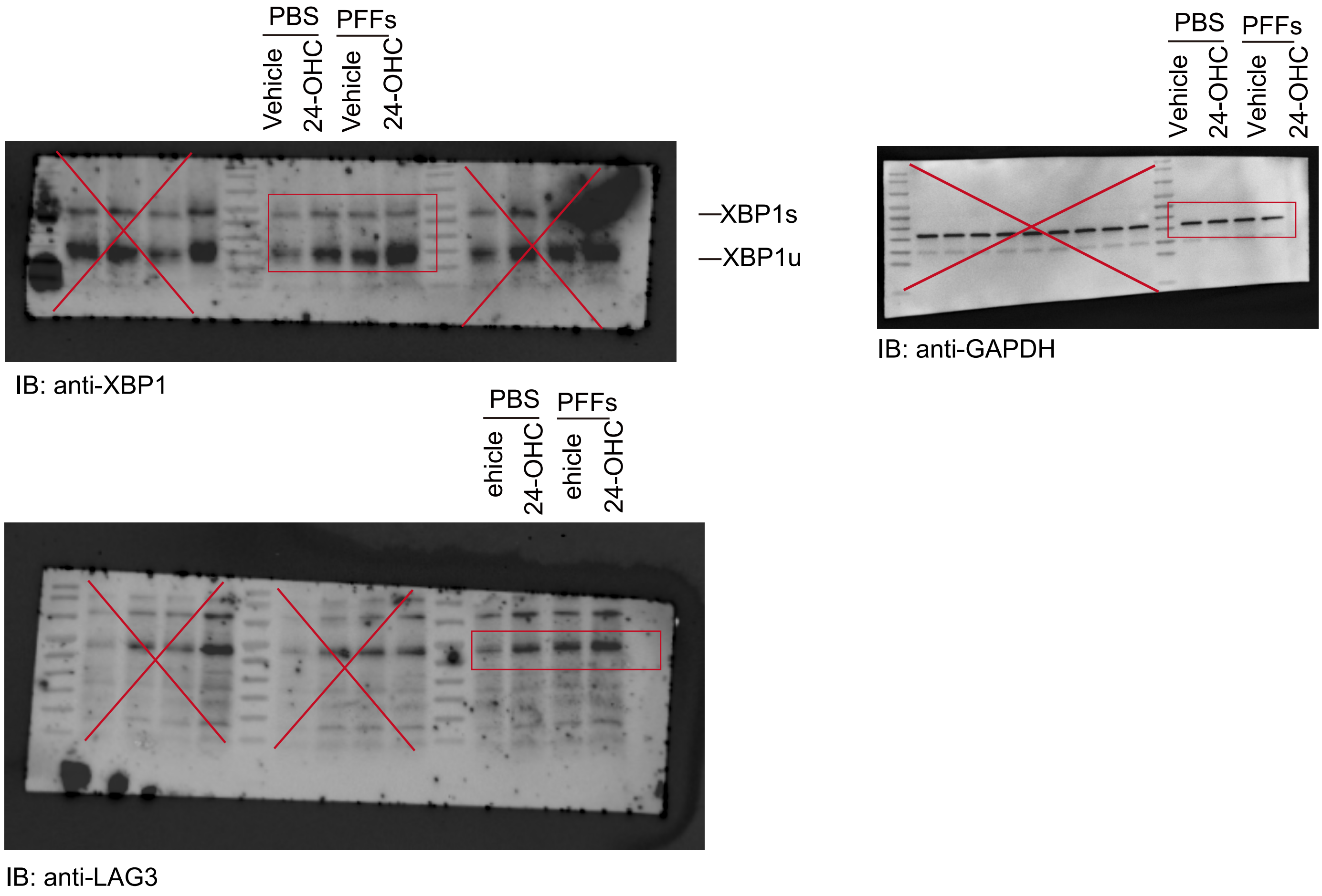

Fig. 7-B

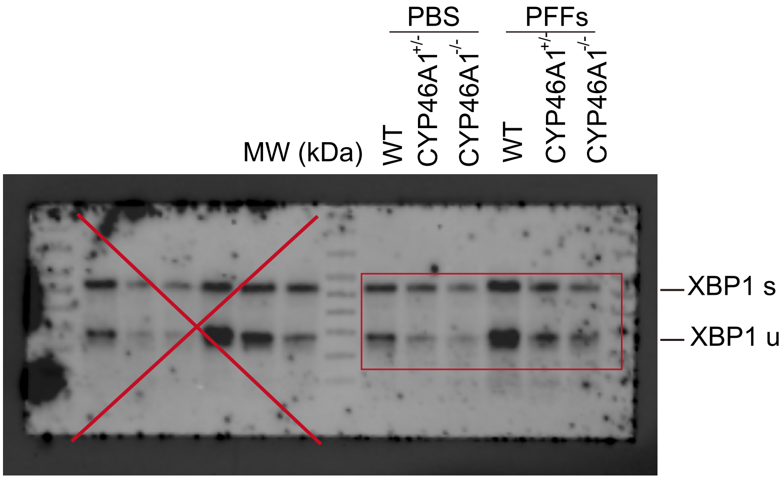

IB: anti-XBP1

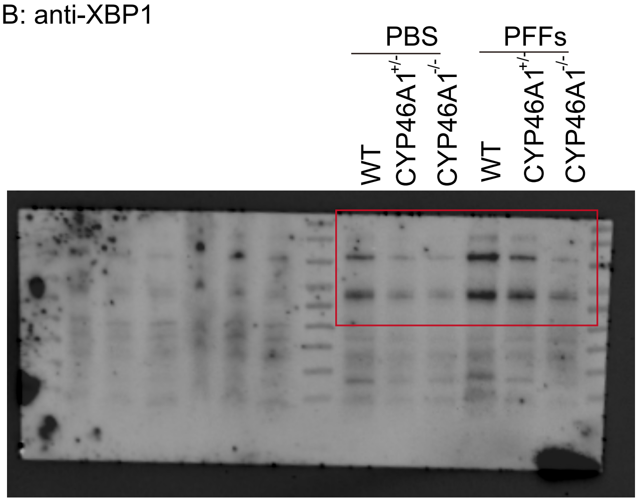

IB: anti-LAG3

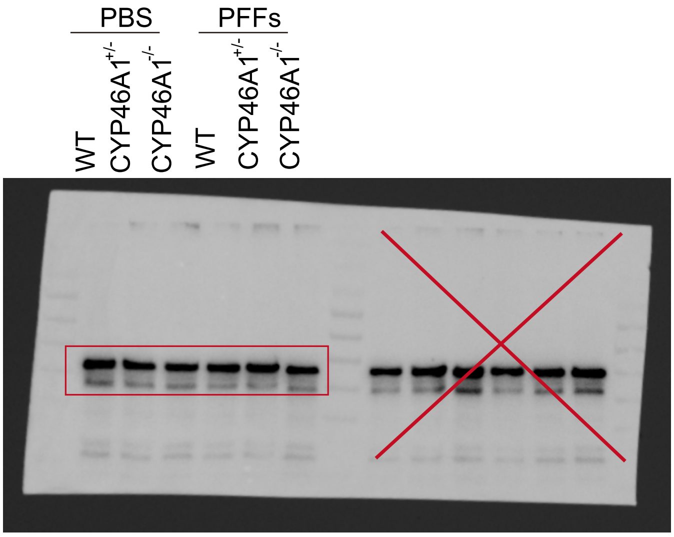

IB: anti-GAPDH

Fig. 7-D

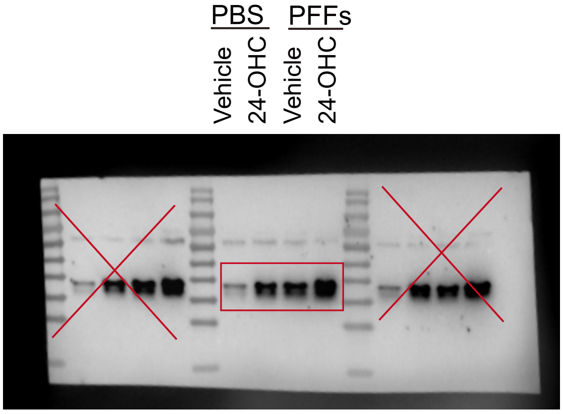

IB: anti-XBP1

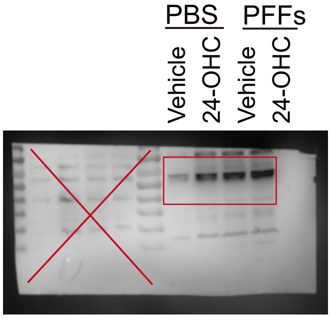

IB: anti-LAG3

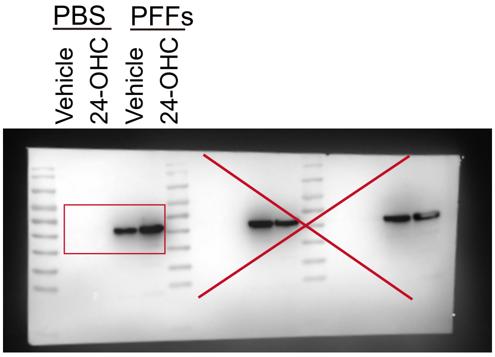

IB: anti-pS129

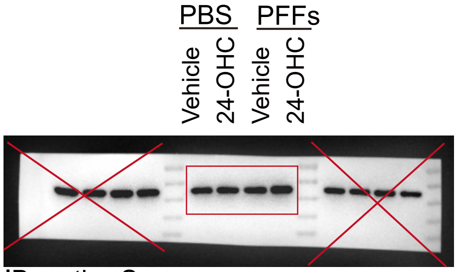

IB: anti-α-Syn

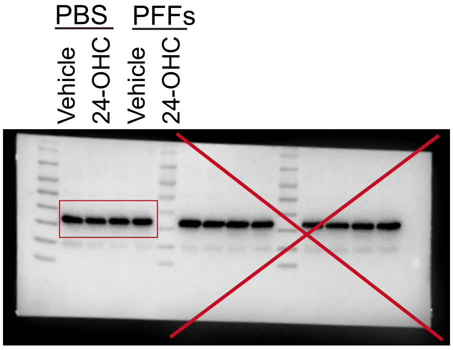

IB: anti-GAPDH

Fig. 7-E

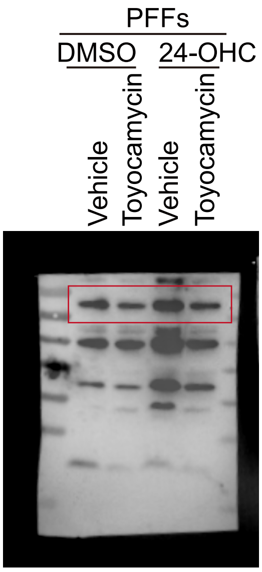

IB: anti-LAG3

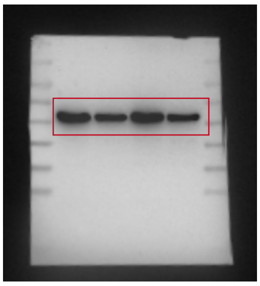

IB: anti-pS129

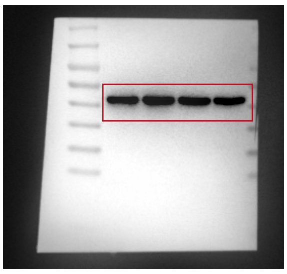

IB: anti-α-Syn

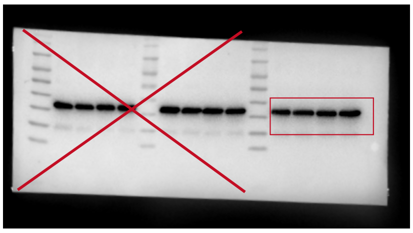

IB: anti-GAPDH

Supplementary Fig.1

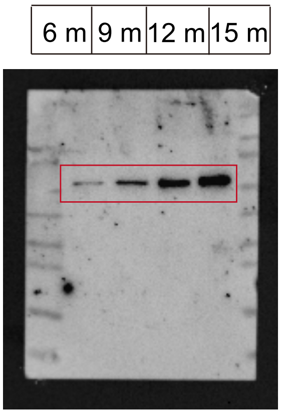

IB: anti-CYP46A1

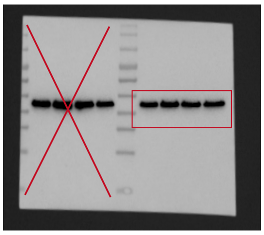

IB: anti-GAPDH

Supplementary Fig. 2-A

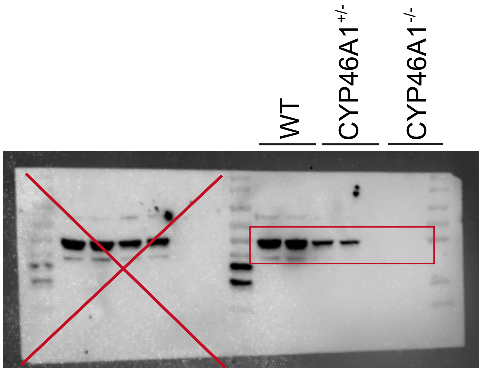

IB: anti-CYP46A1

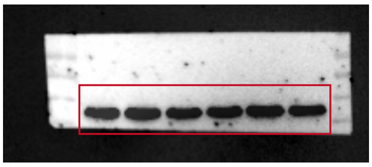

IB: anti-GAPDH

Supplementary Fig. 6-B

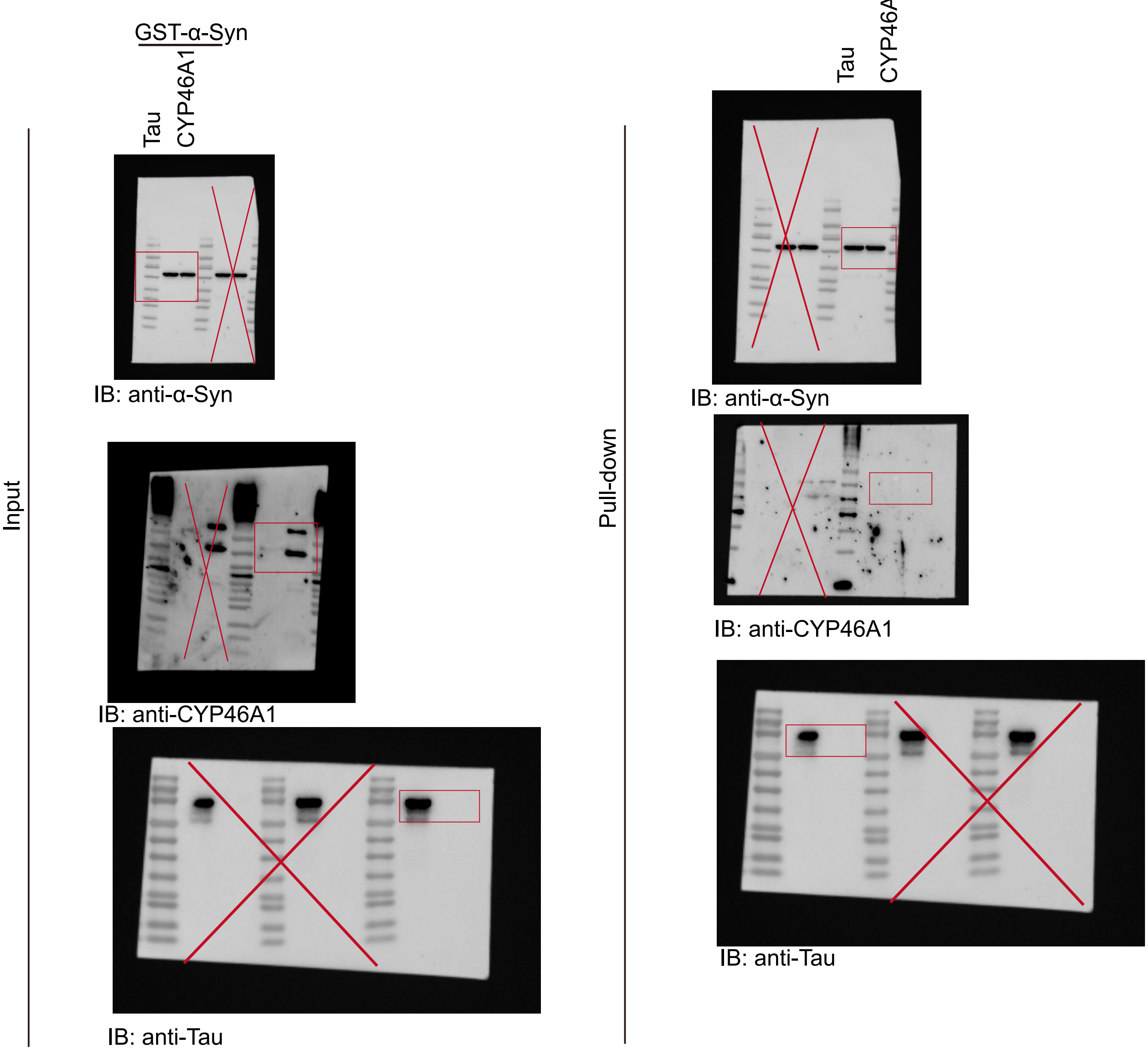

Supplementary Fig. 7-B

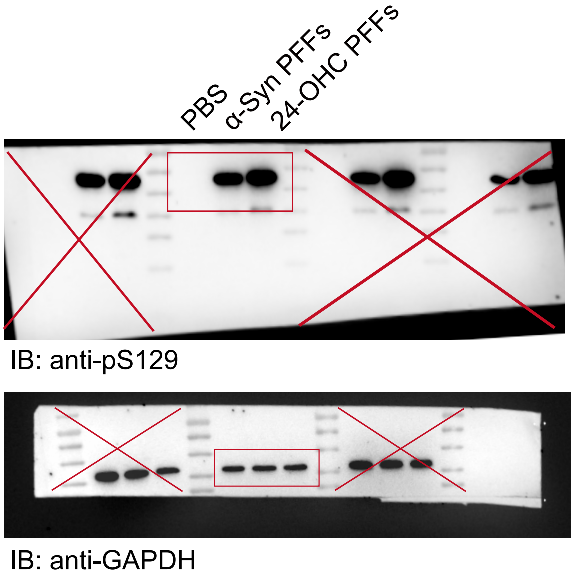

Supplementary Fig. 9-B

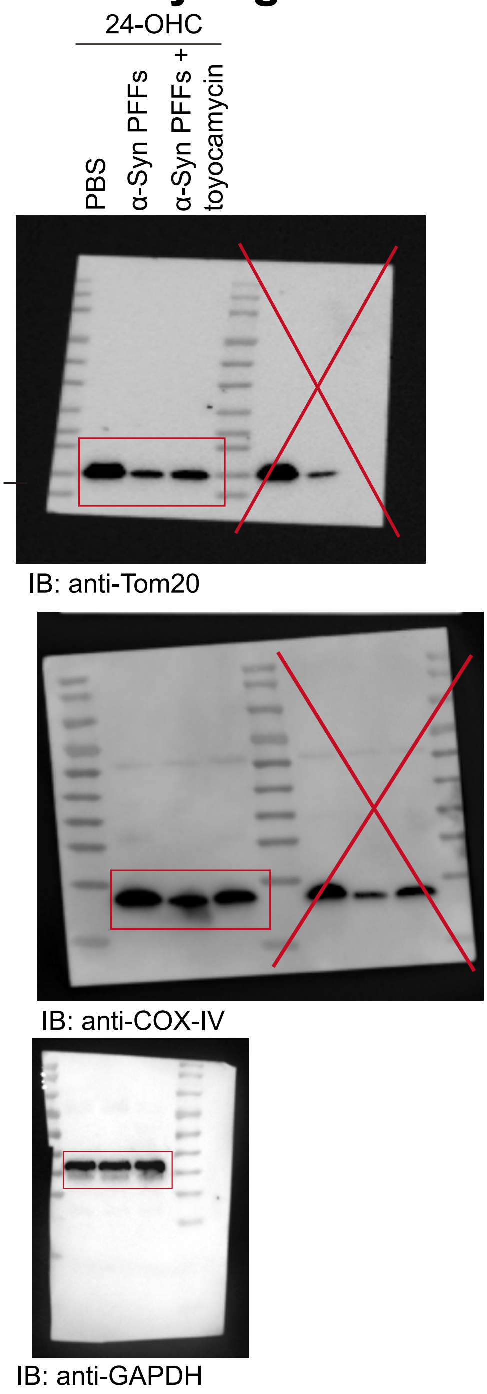

Supplement: S1 Raw Images — (PDF) [file pbio.3002974.s012.pdf]
